# Specificity of mRNA Folding and Its Association with Evolutionarily Adaptive mRNA Secondary Structures

**DOI:** 10.1016/j.gpb.2019.11.013

**Published:** 2021-02-17

**Authors:** Gongwang Yu, Hanbing Zhu, Xiaoshu Chen, Jian-Rong Yang

**Affiliations:** 1 Program in Cancer Research, Zhongshan School of Medicine, The Fifth Affiliated Hospital, Sun Yat-sen University, Guangzhou 510080, China; 2 Department of Medical Informatics, Zhongshan School of Medicine, Sun Yat-sen University, Guangzhou 510080, China; 3 Zhongshan School of Medicine, Sun Yat-sen University, Guangzhou 510080, China; 4 Department of Genetics and Cell Biology, Zhongshan School of Medicine, Sun Yat-sen University, Guangzhou 510080, China; 5 RNA Biomedical Institute, Sun Yat-Sen Memorial Hospital, Sun Yat-sen University, Guangzhou 510120, China

**Keywords:** Evolutionary genomics, RNA secondary structure, RNA folding specificity, RNA alternative folding, Ribosome density

## Abstract

The secondary structure is a fundamental feature of both non-coding RNAs (ncRNAs) and messenger RNAs (mRNAs). However, our understanding of the secondary structures of mRNAs, especially those of the coding regions, remains elusive, likely due to translation and the lack of RNA-binding proteins that sustain the consensus structure like those binding to ncRNAs. Indeed, mRNAs have recently been found to adopt diverse alternative structures, but the overall functional significance remains untested. We hereby approach this problem by estimating the folding specificity, *i.e*., the probability that a fragment of an mRNA folds back to the same partner once refolded. We show that the folding specificity of mRNAs is lower than that of ncRNAs and exhibits moderate evolutionary conservation. Notably, we find that specific rather than alternative folding is likely evolutionarily adaptive since specific folding is frequently associated with functionally important genes or sites within a gene. Additional analysis in combination with **ribosome density** suggests the ability to modulate ribosome movement as one potential functional advantage provided by specific folding. Our findings reveal a novel facet of the RNA structurome with important functional and evolutionary implications and indicate a potential method for distinguishing the m**RNA secondary structures** maintained by natural selection from molecular noise.

## Introduction

Single-stranded RNA molecules spontaneously fold into various secondary structures via intramolecular base pairing. These structures, especially those evolutionarily conserved, are considered essential for the functions of non-coding RNAs (ncRNAs), such as transfer RNAs (tRNAs), microRNAs (miRNAs) [1], small nuclear RNAs (snRNAs) [2], small nucleolar RNAs (snoRNAs) [3], and ribosomal RNAs (rRNAs) [4]. For coding/messenger RNAs (mRNAs), secondary structures are implicated in the localization [5], (de)stabilization [6], [7], and editing [8] of RNA molecules, as well as in the regulation of translational repression [9], [10], translational elongation speed [11], [12], and cotranslational protein folding [13], [14]. Furthermore, natural selection for functional RNA secondary structures constrains the evolution of RNA sequences [15], [16]. Therefore, the study of the RNA structurome is of fundamental importance in RNA biology and evolutionary biology. Recently, the development of high-throughput sequencing (HTS)-based assays for RNA secondary structures, such as parallel analysis of RNA structure (PARS) [17], icSHAPE [18], FragSeq [19], structure-seq [20], RNA proximity ligation (RPL) [21], psoralen analysis of RNA interactions and structures (PARIS) [22], and sequencing of psoralen crosslinked, ligated, and selected hybrids (SPLASH) [23], has started to reveal a more complete picture of the secondary structures of different RNA molecules.

Despite these advancements, our understanding of the secondary structures of mRNAs, especially those of the coding regions, is mostly anecdotal. One major obstacle has been that mRNA molecules are frequently threaded into translating ribosomes, which can only accommodate a single-stranded mRNA. This repeated disruption by translation triggers frequent refolding of mRNAs and presumably makes the experimental detection of a consensus structure difficult *in vivo*. Indeed, the active unfolding of secondary structures has been detected in thermostable mRNAs in yeast, suggesting an incomplete role of thermodynamics in mRNA folding *in vivo* [24]. In contrast, a majority of structured ncRNAs have a single functional secondary structure, which is usually stabilized by protein molecules or other molecules.

In the context of frequent refolding, the physical proximity of two linearly remote fragments within the same molecule due to Watson-Crick base pairing or simply RNA “folding”, could be divided into two types. The first type, in which a fragment of an RNA always pairs up with the same remote fragment once refolded, exhibits specific folding, while the second type, in which an RNA fragment is capable of pairing with different remote fragments when refolded, exhibits non-specific/alternative folding. A classic example of alternative folding is riboswitches, the function of which is dependent on the exchange between two mutually exclusive conformations [25], [26].

Theoretically, mRNA folding should not be more specific than the folding of ncRNAs, because the folding of ncRNAs is usually stabilized by proteins or other small molecules, whereas translating ribosomes interfere with mRNA folding. For example, either counterpart in a particular folding event could be excluded from any base pairing due to occupation by the ribosome. The alternative folding thus formed is likely retained until further disruption by the ribosome, because the kinetics of spontaneous exchange between alternative structures tend to be slow [27]. Additionally, the frequent reorganization of local mRNA secondary structures by translating ribosomes gives mRNAs ample opportunity to sample alternative (sub)optima in the energy landscape, making mRNAs more likely than ncRNAs to adopt alternative folds. Indeed, it has been recently found that approximately 20%–50% of the top 50 mRNAs with the highest numbers of detected secondary structures have at least one pair of alternative structures, some of which are evolutionarily conserved, suggesting that alternative structures are pervasive [22].

Despite the unambiguous evidence for alternative folding of mRNAs, as well as the importance of mRNA secondary structures, there has been a lack of systematic investigations on the folding specificity of mRNAs, let alone its functional and evolutionary significance. In particular, several questions regarding folding specificity are critical to our understanding of RNA biology. For example, what is the average level of folding specificity of mRNAs, and how does folding specificity affect mRNA functions? Theoretically, if the structures of mRNA molecules from the same gene are too diverse, few of these structures can have significant functions due to the negligible number of molecules that adopt each structure. In contrast, isolating foldings with strong specificity should help identify functional structures. From an evolutionary perspective, it is also important to determine whether and why alternative or specific folding is generally adaptive.

To answer these questions, we studied the folding specificity of mRNAs using HTS data for RNA folding in yeast and mouse. We confirmed the reduction of the folding specificity of mRNAs compared to that of ncRNAs and demonstrated the heterogeneity of folding specificity among genes and sites within the same gene. Furthermore, folding specificity, instead of diversity, is highly prevalent in important genes and subgenic regions, suggesting that folding diversity is generally nonadaptive. By demonstrating the different ribosome stalling capacities of specific and non-specific foldings, we provided one potential mechanistic explanation for the functional impact and evolutionary benefit of specific mRNA folding. Finally, we identified unique folding specificity signatures around the 70th nucleotide after the start codon, which is consistent with a known function of mRNA folding in translational control. Altogether, our results revealed a novel facet of the RNA structurome with important functional and evolutionary implications, providing novel insights into mRNA secondary structure.

## Results

### Estimation of the mRNA folding specificity

By definition, the folding specificity of an RNA fragment can be estimated by its probability of pairing up with one or more remote fragments. Qualitatively, if two fragments pair up with 100% probability once refolded, the folding is specific, and vice versa. Quantitatively, the level of folding specificity is determined by the number and relative frequency of alternative foldings, or, in other words, the opposite of the diversity of folding partners. Experimental data regarding folding specificity had not been available until the recent advancement of HTS-based assays for RNA duplexes *in vivo* [28]. In particular, RPL followed by deep sequencing yielded chimeric reads with ligation junctions in the vicinity of structurally proximate bases in yeast [21]. In addition, PARIS employed psoralen crosslinking to globally map RNA duplexes with near-base-pair resolution in mouse cells [22]. With these datasets, a list of folding partners for each RNA fragment can be extracted and used to estimate the folding specificity (see Materials and methods). Notably, both assays have yet to attain base-pair resolution; thus, we studied the specificity of folding instead of pairing herein.

To evaluate the folding diversity based on experimental data, we need a unified measurement that accounts for both the number and relative frequency of alternative foldings. To this end, we borrowed the idea of Shannon’s metric of information entropy (*i.e.*, Shannon index) [29], which quantifies the uncertainty in predicting the identity of a randomly chosen entity from a system. The Shannon index is frequently used as an index of species diversity in ecology (the uncertainty in predicting the species randomly captured from a community) [30], or as an index of similarly for the measurement of molecular diversity in various biological systems (the uncertainty in predicting the sequences randomly picked from a pool of nucleic acids or proteins) [31], [32], [33], [34], or as an index of RNA folding specificity, as defined by the computationally predicted ensemble of secondary structures [35]. Based on the Shannon entropy, we measured the diversity of folding by the equation $\;S_{obs}=\;-\sum_{i,j}p_{i,j}lnp_{i,j}$. Here, *i* and *j* are two nucleotides of the RNA, and $\;p_{i,j}\;$ is the probability that the physical proximity between nucleotides *i* and *j* is observed among all the chimeric reads derived from the gene in the RPL or PARIS assay (see Materials and methods). To avoid the confounding effects of sequencing depth and number of relevant sites, $S_{obs}$ is further compared to its theoretical maximal value $\;S_{max}=\;-ln\left(\frac{1}{n}\right)$, where *n* is the total number of pairs of nucleotides for which the physical proximity is revealed by at least one chimeric read. Here, $S_{max}$ is essentially the information entropy when the folding of all relevant nucleotides is equally supported by the experimental data. The folding specificity is then defined as $\;S=\;S_{max}-S_{obs}$, wherein the higher is the *S* value, the stronger is the folding specificity (Figure S1). The equation for *S* is mathematically equivalent to the Theil index, which is commonly used to measure economic inequality.

**Figure 1:**
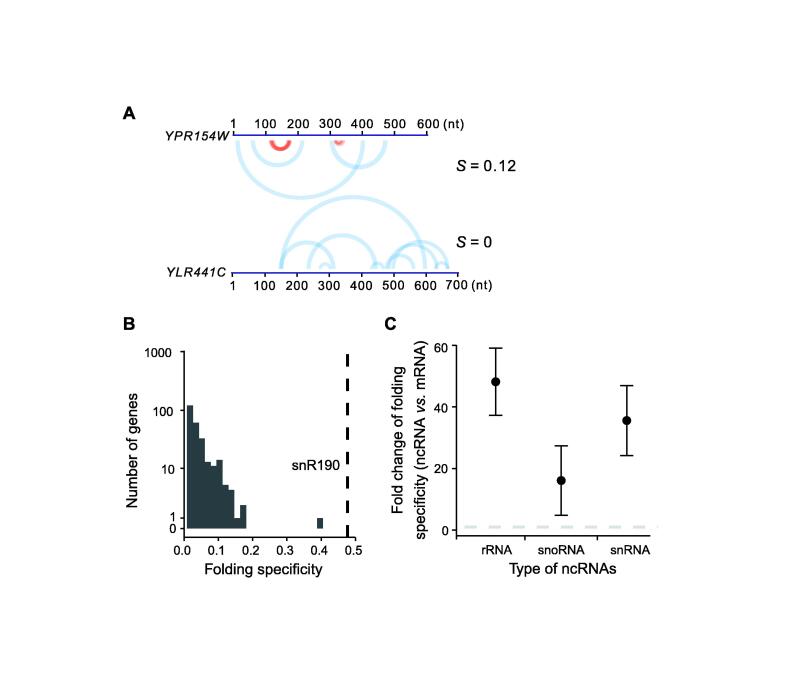
**Folding specificity of yeast mRNAs** **A.** Two examples of folding specificity estimated from RPL data [21]. Two yeast genes with their names, lengths, and corresponding folding specificities (*S*) are shown. Each arch connects the two fragments that are folded together, as suggested by one chimeric read in the RPL data. The red arches support specific folding with minor offsets, whereas the blue arches support non-specific folding. The arches are transparent so that multiple chimeric reads supporting the folding of the same pair of fragments will be visible as deep colors. Specific folding is apparent for *YPR154W* but absent for *YLR441C*, and the folding specificity is thus higher in the former (*S* = 0.12) than in the latter (*S* = 0) (see also Figure S1). **B.** Distribution of the folding specificities of mRNAs in yeast. A total of 697 mRNAs with at least 5 intramolecular chimeric reads are used. The folding specificity of the non-coding snoRNA, snR190, is indicated by a dashed line as a comparison. **C.** Comparison of average folding specificities between the major types of ncRNAs and mRNAs in yeast. The fold change (ncRNA *vs.* mRNA) of 1 is indicated by a horizontal dashed line. Error bars indicate SE, as estimated by bootstrapping the protein-coding genes 1000 times. mRNA, messenger RNA; ncRNA, non-coding RNA; snoRNA, small nucleolar RNA; snRNA, small nuclear RNA; RPL, RNA proximity ligation; SE, standard error.

We first calculated the folding specificity of yeast mRNAs using the RPL data [21]. The recapitulation of folding specificity by *S* was confirmed by manual inspection of certain genes (Figure 1A). For example, in *YLR441C*, none of the experimentally revealed foldings were supported by more than one chimeric read. On the other hand, for *YPR154W*, some of the folds were supported by multiple chimeric reads with minor offsets. The folding specificities of these two open reading frames (ORFs) were respectively quantified as *S* = 0 and 0.12. Similarly, we estimated the folding specificities of all yeast mRNAs with at least 5 chimeric reads (Figure 1B). We found that the folding specificities of yeast mRNAs varied greatly among genes. A substantial number of genes showed no measurable signal for folding specificity, whereas one gene (*YDR420W*) exhibited folding specificity comparable to that of the ncRNA, snR190 (Figure 1B). The mRNA folding specificity distribution was similar when we only used yeast genes with at least 10 chimeric reads (Figure S2A) or used the folding specificities derived from mouse PARIS data (Figure S2B). Additionally, the two biological replicates of the mouse PARIS data allowed us to compare the mRNA folding specificities estimated by the two datasets, and the Pearson’s correlation coefficient (PCC) of the two replicates was 0.48 (Figure S2C; *P* < 1 × 10^−231^), suggesting that the heterogeneity of folding specificity is an intrinsic property of the transcriptome rather than experimental noise.

We also compared the folding specificity of yeast mRNAs with that of several types of ncRNAs. It is well known that ncRNAs can fold into extensive secondary and tertiary structures, which determine the functionality of these ncRNAs. Unlike mRNAs, the secondary structures of ncRNAs are not disrupted by translating ribosomes but are usually stabilized by proteins or other small molecules. Therefore, the folding specificity of ncRNAs is expected to be higher than that of mRNAs. Indeed, the folding specificity of ncRNAs was significantly higher than that of mRNAs (Figure 1C, Figure S2D). Similar results were observed for the mouse PARIS data (Figure S2E and F), highlighting the biological relevance of the folding specificity. Notably, the *S* value is usually low, indicating a weak signal for folding specificity, a phenomenon likely caused by both limited coverage of RPL/PARIS and frequent refolding of mRNAs *in vivo*.

### Folding specificity is moderately stable during evolution

**Figure 2:**
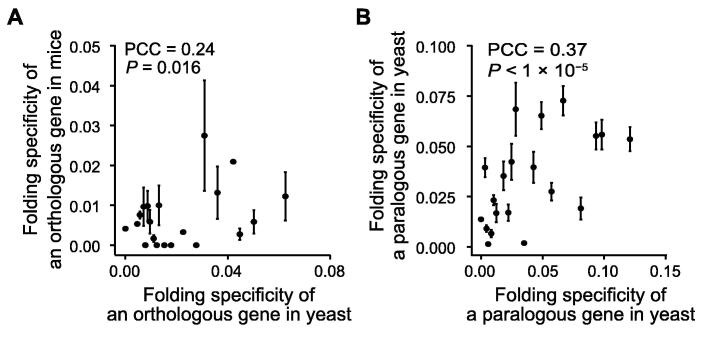
**Folding specificity is moderately stable during evolution** **A.** The folding specificities of one-to-one orthologs between yeast and mouse exhibit a moderate positive correlation. All gene pairs with necessary information are divided into 20 groups based on the folding specificity of the yeast ortholog shown on x-axis. The average folding specificity (mean ± SE) of the mouse ortholog is shown on y-axis. **B.** The folding specificities of paralogous gene pairs in yeast are positively correlated. All gene pairs with necessary information are binned into 20 groups based on the folding specificity of one of the yeast paralogs shown on x-axis. The average folding specificity (mean ± SE) of the other paralog is shown on y-axis. PCC based on unbinned data is also indicated within each panel. PCC, Pearson’s correlation coefficient.

To further assess the biological significance, we compared the folding specificities of one-to-one orthologs between yeast and mouse protein-coding genes, and a moderate yet significant positive correlation was observed (Figure 2A; PCC = 0.24, *P* = 0.016). Considering the experimental noise and technical differences underlying the RNA folding data for yeast (RPL) and mouse (PARIS), the actual correlation should be stronger than observed. Indeed, when we compared the folding specificities of paralogous gene pairs in yeast, an enhanced correlation was observed (Figure 2B; PCC = 0.37, *P* < 1 × 10^−5^). We similarly compared the folding specificities of all pairs of orthologous ncRNA genes with the necessary data from yeast and mouse, including 10 pairs of snoRNAs, 2 pairs of rRNAs, and 1 pair of snRNAs. We observed a strong correlation (PCC = 0.86, *P* = 0.006), even though the sequence conservation of the snoRNAs was so poor that ortholog identification had to be performed based on the corresponding targets [36]. These results suggest that the folding specificity is moderately stable between orthologous gene pairs as well as between paralogous gene pairs, and is thus partially controlled by purifying selection. Together, the aforementioned observations imply that folding specificity is a gene-specific molecular trait with probable functional and evolutionary effects.

### Relationship between folding specificity and thermostability

**Figure 3:**
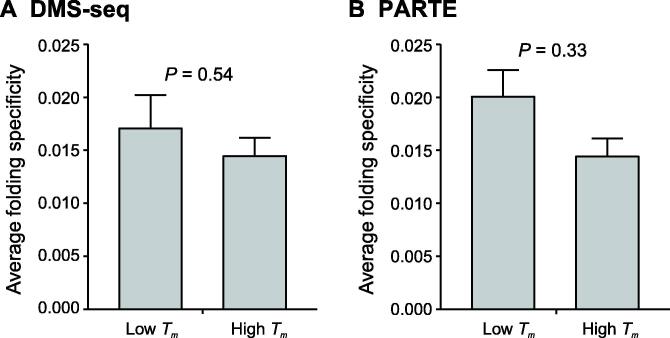
**The folding specificity of mRNA is not dominated by thermodynamics** **A.** Comparison of average mRNA folding specificities between the “low *T_m_*” and “high *T_m_*” groups based on the *T_m_* values determined by the DMS-seq experiment. **B.** Comparison of average mRNA folding specificities between the “low *T_m_*” and “high *T_m_*” groups based on the *T_m_* values determined by the PARTE experiment. In both panels, yeast protein-coding genes are divided into two equal-sized groups, “low *T_m_*” and “high *T_m_*” groups, with high and low average *T_m_* values, respectively. Error bars indicate SE, and *P* values are determined by the Mann-Whitney *U* test. DMS-seq, dimethyl sulfate sequencing; PARTE, parallel analysis of RNA structures with temperature elevation; *T_m_*, melting temperature.

Thermodynamic equilibrium dictates that for any ensemble of RNA molecules with identical sequences, the fraction of molecules folded into a certain structure is exponentially proportional to the thermostability of the structure, *i.e.*, the RNA structure exhibits a Boltzmann distribution. In other words, RNAs have a high probability of folding into thermodynamically stable structures, which might enhance folding specificity. To determine the relationship between folding specificity and thermostability, we obtained the average melting temperatures (*T_m_* values) of the *in vitro* RNA secondary structures for each yeast mRNA, as derived by two different experimental techniques, namely, dimethyl sulfate sequencing (DMS-seq) [24] and parallel analysis of RNA structures with temperature elevation (PARTE) [37] (see Materials and methods). We divided the yeast genes into two equal-sized groups with high or low average *T_m_* values. For both DMS-seq- and PARTE-derived *T_m_* values, we did not observe statistically significant differences in folding specificity between the two groups of genes (Figure 3A and B). These results suggest that the folding specificity of mRNAs is not dominated by the thermostability of mRNAs, consistent with previous observations [38]. The results are also consistent with a model in which frequent unfolding by translating ribosomes, combined with the relatively slow kinetics of exchange between alternative structures [27], causes substantial deviation from the ribosome-free thermodynamic equilibrium. This phenomenon is similar to the emergence of nascent RNA from RNA polymerase, which exhibits sequential folding *in vivo* [39]. Notably, however, thermostability might play a prominent role for ncRNAs and inactively translating mRNAs.

### Important genes have strong folding specificities

Pervasive alternative folding, which is occasionally evolutionarily conserved, has been previously observed [22]. It has been argued that at least some alternative foldings are likely functional [22]. However, it remains untested whether a majority of the alternative foldings or foldings with high diversity, are evolutionarily adaptive. We reasoned that functionally detrimental mutations are more deleterious when they occur on important genes than on other genes; therefore, the adaptive molecular phenotype, be it folding specificity or diversity, should be more constrained by (purifying) natural selection in important genes than in other genes. In other words, folding specificity should be more pronounced in important genes if it is generally adaptive, and vice versa for folding diversity. To test this hypothesis, we compared the folding specificity with different proxies of gene importance.

**Figure 4:**
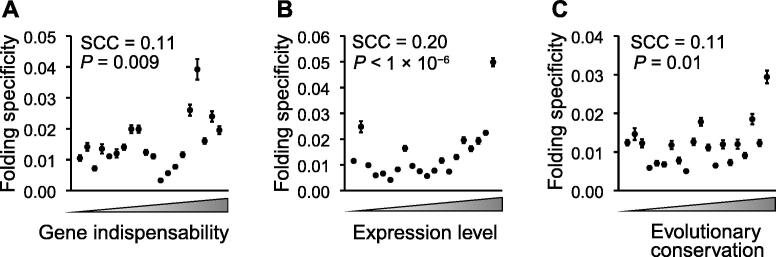
**Important genes have strong folding specificities** The functional importance of a gene is approximated by its gene indispensability (**A**), mRNA expression level (**B**), and evolutionary conservation (**C**). The genes are binned into 20 equal-sized groups based on the rank of the gene in the three proxies of functional importance. The mean folding specificity of each group and the 95% confidence interval assessed by 1000 bootstraps of the genes are indicated by the dots and the corresponding error bars. SCC of the unbinned data is shown in each panel. SCC, Spearman’s rank correlation coefficient.

First, gene importance can be measured by gene indispensability, *i.e.*, the opposite of the organismal fitness upon deleting a gene. We estimated the indispensability of a gene as the negative value of the fitness of a yeast strain that lacks the gene [40] and compared this indispensability with the folding specificity of the corresponding mRNA. As a result, we found that the folding specificity was positively correlated with the gene indispensability [Spearman’s rank correlation coefficient (SCC) = 0.11, *P* = 0.009; Figure 4A], with two-fold higher folding specificities for the 5% most important genes than those for the 5% least important genes. There is no gene indispensability data for mouse. We thus divided the mouse genes into essential and nonessential groups and found that the folding specificities were significantly higher for the essential genes than for the nonessential genes (*P* < 1 × 10^−7^, Wilcoxon rank-sum test; Figure S3A). These results suggest that folding specificity is generally adaptive and that folding diversity is likely nonadaptive molecular or experimental noise.

Second, we used the mRNA expression level as a proxy of gene importance. It is believed that due to the sheer number of mutant molecules, mutations in highly expressed mRNAs exert cytotoxicity that is otherwise negligible in mRNAs with low expression levels [41]. If folding specificity plays a role in the repression of such expression-dependent cytotoxicity, natural selection should have maintained high folding specificity in highly expressed genes. Consistent with the pattern of gene indispensability, we found that the folding specificity was positively correlated with the mRNA expression level in yeast, with the 5% most abundant mRNAs exhibiting four-fold higher folding specificities than those of the 5% least-expressed genes (SCC = 0.20, *P* < 1 × 10^−6^; Figure 4B). This correlation cannot be explained by longer half-lives conferred by specific foldings because the folding specificity is uncorrelated with the mRNA half-life [42] (SCC = 0.017, *P* = 0.659). To further examine whether this pattern is an artifact of the abundance of chimeric reads for highly expressed mRNAs, we randomly sampled five intramolecular chimeric reads from each mRNA and recalculated the folding specificity. This randomized down-sampling was repeated 1000 times, and the resulting folding specificity remained positively correlated with the mRNA expression level (*P* = 0.039, permutation test; Figure S3B). In addition, we compared the folding specificity with the mRNA expression level in mouse and again identified a positive correlation (SCC = 0.28, *P* < 1 × 10^−127^; Figure S3C and D), lending further support to the adaptiveness of folding specificity.

Third, we compared the folding specificity with the evolutionary conservation (see Materials and methods) of the gene. Because highly conserved genes are under strong functional constraints [41], we predicted stronger folding specificities for highly conserved genes than for poorly conserved genes, given the previous observation regarding protein indispensability and expression level. Indeed, we observed a positive correlation between the evolutionary conservation and the folding specificity in both yeast (SCC = 0.11, *P =* 0.01; Figure 4C) and mouse (SCC = 0.14, *P <* 1 × 10^−29^; Figure S3E). In summary, three different proxies of gene importance consistently support the hypothesis that folding specificity is adaptive but, on the other hand, suggest that folding diversity is likely a nonadaptive phenomenon derived from molecular stochasticity or experimental noise.

### RNA circularization cannot fully explain the folding specificity of highly expressed mRNA

**Figure 5:**
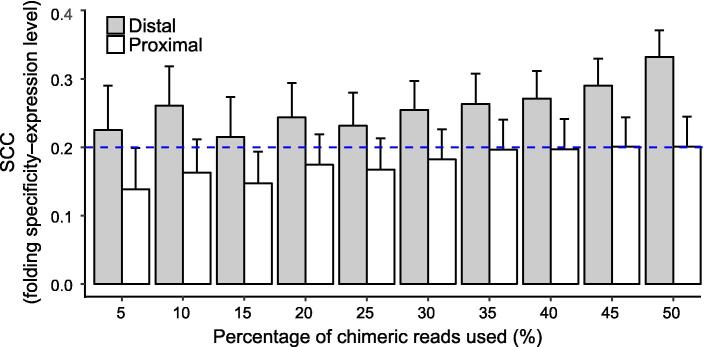
**RNA circularization cannot fully explain the folding specificity of highly expressed mRNA** According to the circularization scores, chimeric reads are categorized into proximal/distal folding. Spearman’s correlations between the mRNA expression level and the folding specificity estimated using different fractions of proximal or distal folding in each gene are shown. Error bars indicate SE, as estimated by bootstrapping the genes 1000 times. The blue dashed line indicates the correlation when all the intramolecular foldings are used.

Hereinafter, we will focus on RPL data from yeast unless otherwise noted because of the availability of various types of functional genomic data in yeast (see below) and the relatively high coverage in yeast for accurate quantification of folding specificity. It has been previously reported that the circularization of mRNAs by eukaryotic translation initiation factors facilitates ribosomal recycling and efficient mRNA translation [43]. Indeed, RNA folding with a long intervening distance is more prevalent in genes with high translational efficiency than those with low translational efficiency [23]. If highly expressed mRNAs are also highly translated [44], the observed correlation between the mRNA expression level and the folding specificity might then be explained by the dominance of long-distance foldings, particularly those connecting the 5′ and 3′ ends of the mRNA. To rule out such a possibility, we calculated the “circularization score” [23] for each RNA folding, which is the distance between the central nucleotides of the two folding partners supported by each chimeric read, normalized to gene length. We then used 5% of the chimeric reads with the top (distal folding) or bottom (proximal folding) circularization scores to recalculate the folding specificity. The correlation between the folding specificity and the mRNA expression level was significantly positive when we used this subset of the data and remained so if we included up to 50% of the distal/proximal folding-related chimeric reads (Figure 5). The aforementioned result indicates that RNA foldings of various distances all contribute to the high folding specificity of highly expressed mRNAs, which thus cannot be explained by RNA circularization. Furthermore, our observation suggests that instead of a local feature limited to a certain fraction of the mRNA sequence [such as the 3′ end, 5′ end, and untranslated region (UTR)], folding specificity impacts the whole mRNA molecule.

### Highly conserved nucleotides fold more specifically than poorly conserved nucleotides within the same gene

**Figure 6:**
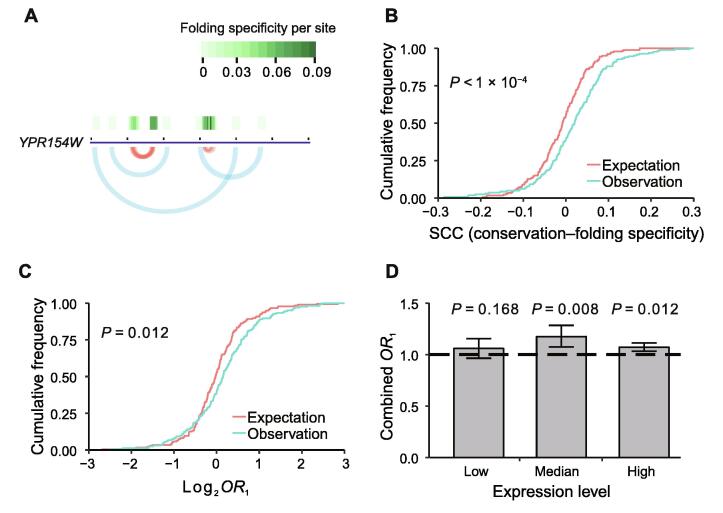
**Strong folding specificity of highly conserved nucleotides within genes** **A.** An example of the folding specificity calculated for each nucleotide. The gene and the arches indicating folding are the same as those in Figure 1A. The per-site folding specificities are further shown as green tiles above the gene and the levels are indicated by the color scale. **B.** Cumulative frequency distribution of the observed and randomly expected within-gene Spearman’s correlations between the evolutionary conservation of each nucleotide and the corresponding folding specificity. The random expectation is generated by shuffling the per-nucleotide conservation level. The *P* value is determined by the Kolmogorov-Smirnov test. **C.** Cumulative frequency distribution for the observed and randomly expected *OR*_1_ values. *OR*_1_ measures the enrichment of nucleotides with high folding specificities at evolutionarily conserved residues within a gene. The random expectation is generated by shuffling the per-nucleotide conservation level. The *P* value is determined by the Kolmogorov-Smirnov test. **D.** Bar chart showing the combined *OR*_1_ values for three groups of yeast genes with low, median, and high expression levels, respectively. The yeast genes analyzed are grouped into three equal-sized bins based on their expression levels, and the combined *OR*_1_ values are estimated by the Mantel-Haenszel procedure. The horizontal dashed line indicates the value of *OR*_1_ = 1. Error bars indicate SE, as estimated by bootstrapping the genes 1000 times. *OR*, odds ratio.

With the aforementioned results showing a positive correlation between the folding specificity and the gene importance, it can be similarly predicted that within the same gene, specific foldings should be associated with important regions of the gene. Furthermore, within-gene comparisons of the functional importance and the folding specificity should be completely free of intergenic confounding factors, such as expression level. To verify this hypothesis, we calculated the folding specificity for each nucleotide of an mRNA using the chimeric reads supporting the folding of the focal nucleotide (Figure 6A; see Materials and methods).

We then reasoned that the functional importance of each nucleotide could be approximated by the evolutionary conservation of the nucleotide, which was estimated using one-to-one orthologs in 6 post-whole-genome duplication (WGD) yeast species (see Materials and methods). The level of evolutionary conservation and folding specificity for each nucleotide were subsequently compared for each of 166 distinct genes with the necessary information. Consistent with our prediction, a positive Spearman’s rank correlation was observed for 101 genes, which was significantly higher than the random expectation of 166/2 = 83 (*P* = 0.006, binomial test). For each gene, we also randomly shuffled the folding specificity of each site and re-evaluated the correlation between the evolutionary conservation and the folding specificity, which served as an *ad hoc* random expectation. Compared with this expected distribution, the real within-gene correlation between the folding specificity and the evolutionary conservation was significantly skewed towards positive values (Figure 6B), suggesting an association between important nucleotides and specific folding and that specific folding is likely more adaptive than non-specific folding.

To further assess the relationship between evolutionary conservation and folding specificity for each gene, we constructed a 2 × 2 matrix for each gene by dividing each site of the gene into one of four groups on the basis of the folding specificity and evolutionary conservation and calculated an odds ratio (*OR*_1_) from the matrix (see Materials and methods). If the specifically folded sites are preferentially located in conserved regions, the *OR*_1_ is >1. We similarly generated a randomly expected *OR*_1_ distribution by shuffling the folding specificities among all the sites within each gene, which was found to be dwarfed by the real *OR*_1_ values (Figure 6C). This result again supports the adaptiveness of specific folding.

To determine whether the conservation level at specifically folded sites was affected by expression level, we divided genes with the necessary information into three groups with low, median, or high expression levels and calculated an overall *OR*_1_ for each group using the Mantel-Haenszel procedure (Figure S4; see Materials and methods). In all groups except the group with low expression levels, the combined *OR*_1_ values significantly exceeded 1 (Figure 6D), suggesting that conserved sites in highly expressed genes exhibit a strong propensity to fold specifically, which is consistent with the strong selection observed for highly expressed genes. Finally, we combined all the genes for an overall *OR*_1_ = 1.09 (*P* < 1 × 10^−4^, Mantel-Haenszel test), which again supports the adaptiveness of folding specificity.

To further exclude the possibility that the observed association between conservation and folding specificity within genes can be explained by the local thermodynamic stability, we calculated another odds ratio (*OR*_2_), indicating the overrepresentation of nucleotides with low *T_m_* values and high folding specificities (see Materials and methods). Regardless of the use of DMS-seq- or PARTE-derived *T_m_* values, *OR*_2_ never exceeded 1 (Figure S5), suggesting a negligible effect of *T_m_* on the per-nucleotide folding specificity. In combination with the results of between-gene analyses, our results offer unequivocal support for an overall adaptive role of folding specificity and suggest a nonadaptive role and, thus, likely molecular or experimental noise for folding diversity.

### Ribosome stalling demonstrates the functional impact of folding specificity

Given the results presented above, we then asked the following question: what is the molecular mechanism that grants a selective advantage to folding specificity? We have shown that the adaptiveness of folding specificity is not dependent on the folding distance (Figure 5), so the functional benefit conferred by specific folding is likely generally applicable to the whole mRNA molecule, instead of being confined to small specific regions such as the 5′ and 3′ ends (*e.g.*, regulation of the initiation rate) or intron/exon borders (*e.g.*, regulation of alternative splicing). Therefore, we chose to test the functional impact of folding specificity on ribosome stalling, a molecular phenomenon that is potentially applicable to the whole coding sequence (CDS) [11]. Nevertheless, this test by no means indicates that ribosome stalling is the only functional benefit provided by specific folding.

**Figure 7:**
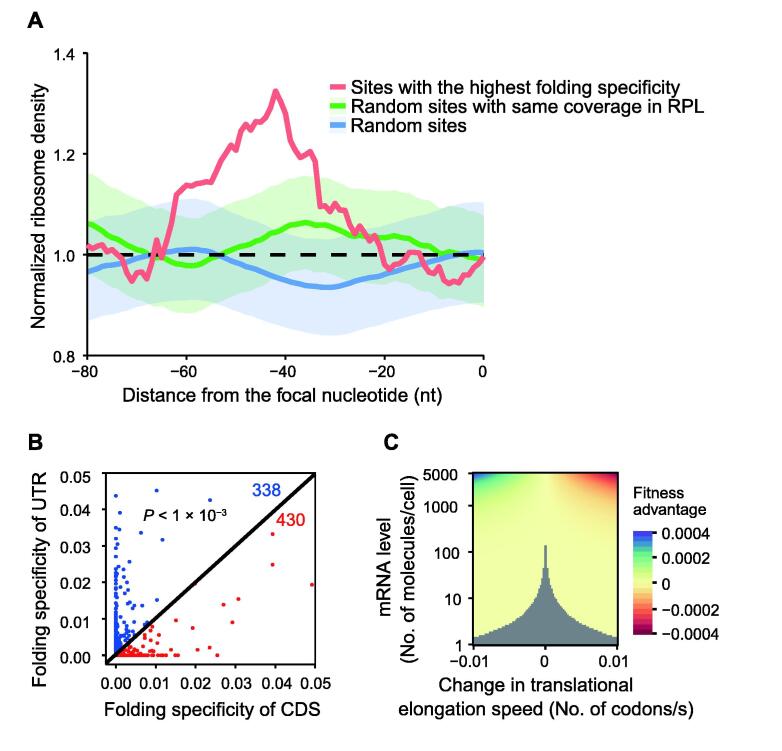
**Effect of folding specificity on ribosome stalling** **A.** Meta-gene analysis of the normalized ribosome density upstream of the most specifically folded nucleotide (*i.e.*, closest to the 5′ end of the mRNA and showing the highest folding specificity) within each gene analyzed. The most specifically folded nucleotide is set at ×  = 0, upstream of which, a peak of ribosome density is shown (red line). As a control, a random site with nonzero coverage in RPL and *S* = 0 is chosen for each gene, and the average normalized ribosome density upstream of this focal site and SE are shown as a blue line and a blue shade, respectively. As another control, a random site with the same RPL coverage as the most specifically folded site and *S* = 0 is chosen for each gene, and the average normalized ribosome density upstream of this focal site and SE are shown as a green line and a green shade, respectively. Neither control exhibits significant deviation from the dashed black line of y = 1. **B.** For each gene with PARIS chimeric reads, the per-nucleotide folding specificity of the CDS is averaged (x-axis) and compared with that of the UTR (y-axis). The dot representing a gene will lie below the diagonal line if the CDS is on average more specifically folded than the UTR (red dot), and vice versa (blue dot). There are significantly more red dots (430) than blue dots (338) compared to the random expectation of (338 + 430)/2 = 384 (*P* < 1 × 10^−3^, binomial test). **C.** Given the influence of folding specificity on ribosome density shown in (A), the fitness effect of mutations that change folding specificity can be estimated by a model that considers ribosome sequestration and translational fidelity (see Materials and methods). If a change in folding specificity can alter the ribosome density of one codon by ∼20%, as shown in (A), the average elongation speed of a gene will change by 0.01 codon/s, which is used as the limit of the x-axis (see Materials and methods). As indicated by the colors, the fitness advantage is shown as a function of the elongation speed (relative to the baseline of 20 codons/s) and gene expression level of the focal gene. The gray area represents fitness effects that are too small to be targeted by natural selection in yeast, the effective population size of which is ∼1 × 10^7^. CDS, coding sequence; UTR, untranslated region.

We have previously shown that ribosome stalling by the mRNA secondary structures modulates translational elongation speed, which is likely utilized by natural selection to balance the trade-offs between translational accuracy and efficiency [11]. Other reports have also suggested the regulatory role of mRNA secondary structures in cotranslational protein folding [14], [45], [46]. We thus asked whether folding specificity affects the capacity of the mRNA secondary structures in the stalling of upstream ribosomes. To this end, we analyzed the local ribosome density upstream of the focal nucleotide that was closest to the 5′ end of the mRNA and showed the highest folding specificity within each gene, using the expression-normalized local Ribo-Seq coverage data (see Materials and methods). We averaged the normalized ribosome densities near the specifically folding nucleotides across genes and found a significant increase in the ribosomal density upstream of the site with specific folding. In particular, we found a maximum of 32% increase in the ribosome density at the 42nd nucleotide upstream of the most specifically folded nucleotide (Figure 7A, red line). This magnitude of ribosome stalling by mRNA folding is comparable to that observed in previous reports [11], [47]. The position of the peak ribosome density likely reflects the limited resolutions of RPL [21] and Ribo-Seq [48], but cannot be explained by the 5′ ramp of translational elongation speed [49], because all specifically folded nucleotides are at least 200 nucleotides downstream of the translational start site.

To assess the expected ribosome density around a random nucleotide captured by RPL, we repeated the local ribosome density analysis using sites with non-specific folding (*i.e.*, *S* = 0) and found no significant increase in the ribosome density upstream of the focal non-specific site (Figure 7A, blue line and blue shade). To further elaborate the effects of specific folding *vs.* non-specific folding, we repeated the local ribosome density analysis with sites that had the same number of chimeric reads as the specifically folded sites and *S* = 0 (Figure 7A, green line and green shade). This additional control again showed no significant increase in the ribosome density upstream of non-specific sites, suggesting an exclusive association of the folding specificity with upstream ribosome stalling.

In addition to ribosome stalling by specific mRNA folding, an alternative explanation for the aforementioned observation is that ribosomes limit the folding choices of flanking nucleotides by steric hindrance, effectively increasing the folding specificity. However, this alternative explanation would predict peaks for Ribo-Seq reads both upstream and downstream of the specifically folded sites, as well as no skewing of the folding specificity due to gene importance. Since both predictions contradict the empirical observations (Figure 4, Figure S6), this result supports the role of folding specificity in the ribosome stalling capacity of mRNA secondary structure. Notably, this result suggests that highly expressed genes, which exhibit increased folding specificity (Figure 4B), have strong control over ribosome velocity. This feature of highly expressed genes is consistent with the increased requirement of these genes for translational fidelity [11] and/or cotranslational protein folding accuracy [50].

To further validate the functional role of folding specificity, we calculated the average per-nucleotide folding specificity of the CDS and that of the UTR for each gene. The mouse PARIS data were used here because UTR annotation is rare in yeast. If folding specificity is involved in the regulation of translation by the ribosome stalling capacity, we shall predict that the average folding specificity of the CDS should be higher than that of the UTR because translation occurs only on the CDS. Consistent with our prediction, we found that for a majority of mouse mRNAs, the folding specificity of the CDS is stronger than that of the UTR (*P* < 1 × 10^−3^, binomial test; Figure 7B), which supports our hypothesized association between folding specificity and translation.

Finally, we asked if the ability of specific folding to slow down elongation is strong enough to make this folding a target of natural selection. To this end, we used a previously published model [11] that incorporates experimentally determined parameters in yeast to predict the effect of changes in the translational elongation speed on fitness. This model considers two competing selective pressures, one for increased elongation speed, which reduces ribosome sequestration, and the other for reduced elongation speed, which increase translational accuracy [11] and reduces protein misfolding [51] (see Materials and methods). We found that a mutation that induced specific folding and increased the ribosome density on one codon by 20% improved the fitness of the cell by 0.03%, when the mutation occurred on highly expressed genes (5000 molecules/cell; Figure 7C, top). Because 3 × 10^−4^ greatly exceeds the inverse of the effective population size (1 × 10^7^) of yeast [52], such a mutation can be targeted by natural selection. Moreover, we found that for genes expressed at low levels (1 molecule/cell), the same mutation only corresponded to a fitness effect of *s* = 7.0 × 10^−8^ (Figure 7C, bottom), effectively making it a neutral mutation (*s* < 1 × 10^7^). In other words, the selection for folding specificity on important or evolutionarily conserved nucleotides should be significant in highly expressed genes but not in genes expressed at low levels, which was exactly what we observed (Figure 6D). Interestingly, this result also suggests that there should be no selection for specific folding in human because even in highly expressed genes, the selective coefficient for enhanced folding specificity (3 × 10^−4^) is much lower than the inverse of the human effective population size (∼1 × 10^3^) [53], which was exactly what we observed using folding specificities derived from human PARIS data [22] (data not shown).

Overall, this result suggests that the folding specificity of an mRNA can be a potential target of natural selection depending on the mRNA expression level, which corroborates the aforementioned observation of weaker folding specificities for genes with low expression levels than those for genes with high expression levels.

### Folding specificity highlights known functional secondary structures

**Figure 8:**
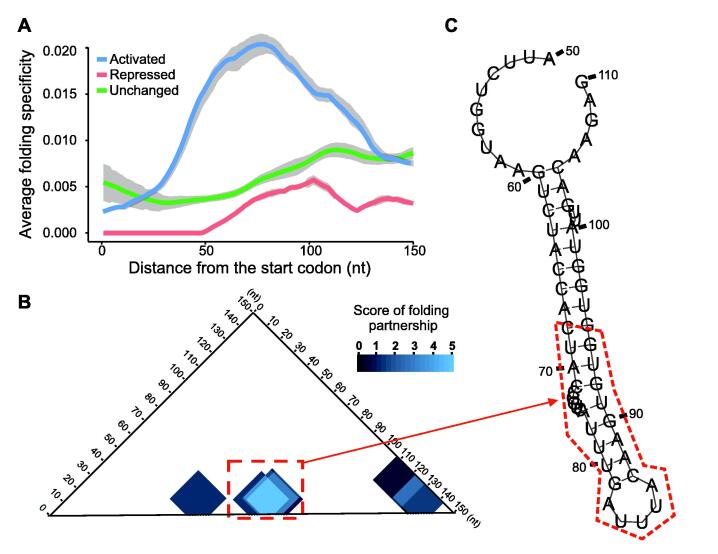
**Elevated folding specificity is consistent with the known functional secondary structure of the CDS** **A.** Meta-gene analysis of the per-nucleotide folding specificity for the first 150 nucleotides after the start codon within mRNAs translationally activated by Dhh1. A region with elevated folding specificity (blue line) is revealed around the 70th nucleotide, which is consistent with previously identified secondary structures that regulate translational initiation by interaction with Dhh1 [54]. The same analyses are conducted as controls for two subsets of mRNAs which are translationally repressed by Dhh1 (red line) or not affected by the loss of Dhh1 (green line). Neither of controls displays a peak of folding specificity near the 70th nucleotide. Each of the three lines shows the mean per-nucleotide folding specificity for the respective subset of genes. The gray shade indicates SE of the mean. **B.** and **C.** An example of the folding specificities of functional secondary structures in the yeast gene *YBR118W*. The scores of folding partnerships (see Material and methods) among the first 150 nucleotide chimeric reads are plotted as color gradients, the scale of which is indicated by the color scale bar (B). The secondary structure predicted within in the region of 50th–110th nucleotides after the start codon of *YBR118W* (C), with constraints derived from the RPL chimeric reads, is also consistent with the existence of a hairpin structure that regulates translation by interaction with Dhh1. Dhh1, DEAD-box RNA helicase.

To further demonstrate the functional relevance of folding specificity, we examined other known functions of secondary structures in CDSs. A previous study has identified a region with a strong secondary structure around the 70th nucleotide after the start codon for a subset of yeast mRNAs [54]. This hairpin structure near the 5′ end of the CDS regulates translation by repressing translational initiation unless the DEAD-box RNA helicase (Dhh1) directly binds and resolves the hairpin [54]. As predicted based on the functional relevance of folding specificity, the per-nucleotide folding specificity of this region should be elevated in an mRNA activated by Dhh1 compared to the folding specificities of other genes. Indeed, we observed a signature increase in the folding specificity of the region around the 70th nucleotide after the start codon (Figure 8A). Taking the transcript *YBR118W* as an example, base pairing within the region of 68th–94th nucleotides is highly specific because of the multiple supportive chimeric reads (Figure 8B). We further predicted the secondary structure within the region of 50th–110th nucleotides after the start codon of the *YBR118W* ORF using RNAfold [55], with structural constraints inferred from the RPL reads. The specific folding appeared as a hairpin near the 70th nucleotide (Figure 8C), which is compatible with the known translational control mechanism [54].

To obtain additional support for the functional relevance of folding specificity, we used a recently published PARIS dataset for the Zika virus [56] to calculate the folding specificity of the viral RNA genome. We observed highly specific folding between the 5′ UTR and the E protein-coding region in the ZIKV PRVABC59 strain (Figure S7). This folding partnership is consistent with a secondary structure that contributes to the infectivity of the Zika virus [56]. Taken together, these results again suggest an association between folding specificity and functional secondary structures.

## Discussion

In this study, we utilize recently published HTS data for RNA duplexes to estimate the specificity of mRNA folding. Consistent with disruption by the translating ribosome, we find that the folding specificity of mRNAs is significantly lower than that of ncRNAs. Unexpectedly, the folding specificity is not stronger for secondary structures with high thermostability *in vitro*. We further observe a positive correlation between the folding specificity and the functional importance among genes and sites within the same gene, suggesting an evolutionarily adaptive role of specific folding. To determine the molecular function underlying the benefit of specific folding, we compare nucleotides associated with specific and promiscuous foldings, and reveal the capacity of ribosome stalling for specific but not promiscuous folding. Finally, we demonstrate the functional relevance of folding specificity via the association of specificity with a translational regulatory hairpin structure among genes activated by Dhh1 in yeast [54] and a previously identified secondary structure contributing to the infectivity of the Zika virus [56]. Our results collectively demonstrate the evolutionary and functional significance of folding specificity and offer new insights for the study of mRNA secondary structures.

One potential caveat in our analyses is that the estimation of the folding specificity might be confounded by the number of short reads that support any folding partnerships within each gene. We incorporate multiple measures to avoid biases created by these potential confounding factors. First, the observed level of folding diversity ($S_{obs}$) is compared with the theoretical maximal value ($S_{max}$) to yield the value of the folding specificity $\;S=\;S_{max}-S_{obs}$, where $S_{max}$ serves as a control for the coverage of RPL/PARIS reads. Second, we conduct a down-sampling analysis for yeast RPL data (Figure S3B) and mouse PARIS data (Figure S3D). Such a down-sampling analysis eliminates the effect of sequencing depth while still revealing a significant positive correlation between the folding specificity and the expression level. Third, our within-gene analysis is free of the aforementioned confounding factors because each nucleotide in the same gene has exactly the same “expression”. These forms of evidence collectively suggest that the correlation between functional importance and folding specificity is robust, regardless of confounding factors such as RNA abundance and sequencing depth. In contrast, the limited resolution of the yeast RPL data and mouse PARIS data, as well as the technical/organismal difference between them, potentially adds random noise to the actual biological signal, which is thus likely stronger than that shown in our study.

The level of RNA folding specificity is expected to be influenced by RNA-binding proteins. For ncRNAs, proteins likely stabilize the native functional RNA folding and thus increase folding stability. In contrast, mRNA folding is expected to be constantly disrupted by translating ribosomes. Indeed, RNA folding occurs on a microsecond scale [57], which is much faster than the *in vivo* translational elongation rate of <30 codons/s [58], [59]. Therefore, mRNA regions that are not occupied by ribosomes have enough time to form local secondary structures, which should change as ribosomes move. The maintenance of specific folding in the face of such frequent disruption suggests tight regulation of the structure, which is therefore likely functional.

Assuming thermodynamic equilibrium, RNA folds into various secondary structures with probabilities dictated by the folding energies. It is, therefore, possible that the folding specificity simply reflects the thermostability of the RNA molecule. In contrast to this possibility, we find no correlation between the folding specificity and the average melting temperature [37] of the RNA secondary structure of a gene. As an additional support for our finding, a previous study on RNA design has already shown that stability and specificity are not closely related [38]. The discrepancy between folding specificity and thermostability may be explained by the effect of translation on mRNAs, wherein ribosomal occupation allows sequential local folding but excludes thermodynamically favored global folding. This phenomenon is similar to the emergence of nascent RNA from RNA polymerase, which has been shown to involve sequential folding *in vivo* [39].

The diversity of RNA folding, as inversely approximated from the folding specificity, is evolutionarily nonadaptive according to comparison with the functional importance of genes or sites within the same gene. This finding is consistent with a model in which molecular stochasticity, a largely nonadaptive intrinsic property underlying all biological processes, has been selectively reduced for important genes. This model is supported by multiple biological phenomena at the molecular level, such as protein expression noise, misinteraction, and misfolding [50], [60], [61], [62], [63], [64], [65]. According to this model, the alternative RNA secondary structure, especially for mRNAs, is likely nonadaptive and selectively constrained by purifying selection against molecular stochasticity. Notably, however, a small fraction of alternative foldings, especially those with relatively high folding specificities, might still be conserved and functional [22].

We find intergenic and intragenic evidence for the evolutionary adaptiveness of folding specificity. There are several hypotheses regarding the exact functional benefit provided by specific mRNA folding that warrant discussion here. First, Mao and colleagues [66], based on computational simulations, have proposed that strong mRNA folding without ribosomes slows down the first translating ribosome, thereby shortening the distance between subsequent ribosomes, eliminating secondary structures in the translating mRNA with increased ribosomal occupancy, and effectively increasing the translational efficiency. In this model, specific folding might play an important role in slowing down the first translating ribosome. However, we find this model to be paradoxical, because slowing down the first translating ribosome is expected to decrease, not increase, translational efficiency. Moreover, it has been estimated that there is, on average, one ribosome per 156 nucleotides of coding regions [67], leaving more than enough nucleotides available for the folding of the translating mRNA, given that the rate of RNA folding (in microseconds [57], [68]) is much faster than that of ribosomal translocation (<30 codons/s [58], [59]). Indeed, the RNA duplexes identified by RPL [21] and PARIS [22] are consistently rich in secondary structures of the translating mRNAs. Therefore, this model is unlikely an explanation for the functional benefit of specific folding.

In another hypothesis, Qi and Frishman [69] have proposed that RNA secondary structures with high and low thermostabilities are under evolutionary pressure to preserve RNA secondary structures and primary sequences, respectively. This model might partially explain the functional benefit provided by specific folding if folding specificity is correlated with thermostability. However, we find that genes with stronger folding specificities are not thermodynamically more stable. Therefore, this model cannot provide a mechanistic link between folding specificity and evolutionary adaptiveness.

A third hypothesis is that specific RNA folding serves as a molecular brake on translating ribosomes, thus enhancing the fidelity of translation and/or cotranslational protein folding [11], [46], [51], [70], [71]. Indeed, it has been suggested that mRNA structure acts as a gauge of cotranslational protein folding by reducing ribosome speed when extra time is needed by the nascent peptide to form and optimize the core structure [14]. This model is compatible with a considerable body of experimental evidence indicating that synonymous variants capable of (de)stabilizing mRNA secondary structures can dramatically alter translation speed and influence cotranslational protein folding [72]. According to our results, specific folding is more efficient than promiscuous folding at regulating ribosome speed. In contrast, it has been recently argued that the ribosome is a major remodeler of RNA structure but RNA structure does not constrain ribosome movement *in vivo* [73]. We propose that the debate over the causal role of ribosome movement *vs.* mRNA structure might be resolved by our observation that only specific folding constrains ribosome movement, whereas non-specific folding is easily unwound by ribosomes.

The mechanism underlying this effect of folding specificity remains to be elucidated, because specific and non-specific foldings should be similar obstacles for ribosome movement, especially when specific folding is not thermodynamically more stable than non-specific folding. One potential explanation is that specific folding is more resistant to the helicase activity of ribosomes [74] than is non-specific folding *in vivo*, such that the resolution of specific folding requires extra time. Nevertheless, regardless of the molecular mechanism, the increased folding specificity of highly expressed genes is consistent with the strong tendency of these genes to avoid mistranslation [11], [75] and misfolding [50], which imposes an expression-dependent fitness cost [75].

Although our study mostly focuses on the folding specificity of coding regions in mRNAs, folding specificity could also be associated with the functional interactions of UTRs. We use a recently published PARIS dataset of the Zika virus [56] and calculate the folding specificity of the genomic RNA of the virus. It has been previously found that a long-range interaction between the 5′ UTR and the E protein-coding region in the Zika RNA genome contributes to infectivity. In support of the association between folding specificity and functional secondary structures, we observe significantly elevated folding specificities for both partners of this long-range interaction (Figure S7).

While significant advances in high-throughput experimental techniques have enabled the dissection of RNA secondary structures on the transcriptomic scale, the extraction of functionally relevant RNA foldings has remained challenging, especially for mRNAs, the folding of which is constantly disrupted by translating ribosomes. The positive correlation between the folding specificity and the functional importance among genes and sites within the same gene, as shown in the current study, indicates a novel strategy involving the prioritization of mRNA structures based on the folding specificity. Indeed, we show ribosome stalling upstream of nucleotides with specific folding but not those with promiscuous folding. We also reveal the elevated folding specificity of the secondary structures implicated in the regulation of translational initiation by Dhh1. These examples demonstrate the usefulness of folding specificity in distinguishing functional and nonfunctional RNA structures.

In summary, the folding specificity of RNA secondary structures and the application of this concept reveal previously unappreciated complexities underlying RNA secondary structures *in vivo*. Specific folding of mRNAs, despite frequent disruption by translating ribosomes, is selectively maintained and is associated with evolutionarily adaptive molecular functions such as the regulation of cotranslational protein folding. An understanding of folding specificity shall provide valuable information for the functional study of RNA secondary structures, particularly for mRNAs.

## Materials and methods

### Genome, annotation, and comparative genomic data

The genomes and annotations were obtained from Ensembl release 89 [76], and the specific genome versions are R64-1-1 for *Saccharomyces cerevisiae* and GRCm38 for *Mus musculus*. The lists of one-to-one orthologs between the two species were also downloaded from Ensembel. Each gene was represented by its longest annotated transcript. To estimate the evolutionary rates of *S. cerevisiae* mRNAs, we also collected mRNA sequences from five other post-WGD yeast species (*S. paradoxus*, *S. mikatae*, *S. bayanus*, *Candida glabrata*, and *S. castellii*), along with gene orthology/paralogy information for these six species from the Fungal Orthogroups Repository [77]. The orthologs between yeast and mouse snoRNAs were extracted from the snOPY database [36], wherein the target genes of the snoRNAs were used to identify orthologs.

### HTS data for RNA folding and estimation of the folding specificity

We used datasets derived from two distinct experimental techniques to assess RNA folding. On the one hand, the single-nucleotide folding anchors for each folding partner derived from the RPL assay [21] in the yeast *S. cerevisiae* were downloaded from the NCBI Gene Expression Omnibus (GEO: GSE69472) [78]. Only the intramolecular folding pairs were retained for further analysis. On the other hand, the raw reads from PARIS [22] for mouse were downloaded from GEO (GEO: GSE74353). The raw reads from PARIS for the Zika virus were downloaded from the European Nucleotide Archive (ENA: PRJEB28648) [56]. The raw reads were then processed using analytical pipelines provided by Lu et al. (https://github.com/qczhang/paris) [22] to yield a list of folding partners. Briefly, short reads were adaptor-trimmed and merged and then aligned to the genome (mm10) by STAR aligner [79]. The reads mapped with gaps or chiastically mapped were combined and assembled into duplex groups by a two-step greedy algorithm, as implemented by the scripts provided by Lu and colleagues [22]. Finally, short reads were extracted from intramolecular duplex groups, and the nucleotides which were located in the center of the 5′ or 3′ fragment and mapped to either folding partner were used as anchors for folding partnership.

Due to the limited resolution, it is difficult to locate the exact pairing partner from RPL or PARIS data. Following the bioinformatics analyses in the RPL assay [21], we instead generated contact probability maps using anchors of the folding partnership derived from either RPL or PARIS. Briefly, using the Python scripts of RPL provided by Ramani and colleagues [21], we computed the coverage at each base of *i* and *j* ($c_{i};c_{j}$), and generated a normalized matrix $M_{\mathrm{ij}}^{norm}=M_{\mathrm{ij}}/\sqrt{c_{i}c_{j}}$, where $\;M_{\mathrm{ij}}$ is the number of reads supporting a folding partnership between *i* and *j*. We then used this matrix to generate M* by binning normalized scores as $M_{\mathrm{ij}}^{*}=\sum_{a=i-10}^{i+10}\sum_{b=j-10}^{j+10}M_{\mathrm{ab}}^{norm}$. Effectively, this is a score of folding partnership for the 21 nucleotides (anchor ± 10 nucleotides) linearly surrounding any pair of folding anchors.

On the basis of $M_{\mathrm{ij}}^{*}$, we can calculate the folding specificity for a whole gene. Based on the Shannon entropy [35], we first calculated $\;S_{obs}=\;-\sum_{i,j}p_{i,j}lnp_{i,j}$ for gene *g*. Here, $p_{i,j}$ is the relative folding probability between nucleotides *i* and *j*, or, in other words, $p_{i,j}=M_{\mathrm{ij}}^{*}/\sum_{g}M^{*}$, with $\sum_{g}M^{*}$ being the sum of all $M_{\mathrm{ij}}^{*}$ within *g*. To avoid the confounding effect of sequencing depth, we also calculated the theoretical maxima of $S_{obs}$ as $\;S_{max}=-\;ln\left(\frac{1}{n}\right)$, where *n* is the total number of pairs of nucleotides of which the physical proximity is revealed by at least one chimeric read. Here, $S_{max}$ equals the information entropy when the folding of every relevant base is equally supported. We then calculated folding specificity as $\;S=\;S_{max}-S_{obs}$, wherein the higher is the *S* value, the stronger is the folding specificity. The equation for *S* is mathematically equivalent to the Theil index, a commonly used metric for economic inequality.

Similarly, we can calculate *S* for an unnecessarily continuous region of a gene by defining $p_{i,j}=M_{\mathrm{ij}}^{*}/\sum_{r}M^{*}$, with $\sum_{r}M^{*}$ being the sum of all $M_{\mathrm{ij}}^{*}$ within *r*. We also calculated the folding specificities for individual nucleotides by defining $p_{i,j}=M_{\mathrm{ij}}^{*}/\sum_{i}M^{*}$, where *i* is the focal nucleotide under study and $\sum_{i}M^{*}$ is the sum of all $M_{\mathrm{ij}}^{*}$ involving *i*.

### Thermostability of mRNA secondary structures

We estimated the thermostability of yeast mRNA secondary structures using data from two experimental techniques, namely, PARTE [37] and DMS-seq [24]. In the PARTE experiment, the footprinting of double-stranded RNA residues by RNase V1 across five temperatures (from 23 °C to 75 °C) was coupled with HTS to reveal the energetic landscape of the transcriptome [37]. In the DMS-seq experiment, the modification of unpaired adenine and cytosine by DMS was monitored by deep sequencing. DMS-seq was used to evaluate the *in vitro* thermostability of RNA folding using genome-wide assays at five temperatures (from 30 °C to 95 °C) [24]. In both PARTE and DMS-seq experiments, the RNA secondary structure unfolds as the temperature increases, allowing estimation of *T_m_*.

We downloaded PARTE and DMS-seq data from GEO (GEO: GSE39680 for PARTE data and GSE45803 for DMS-seq data). The raw reads for both datasets were adaptor-trimmed and mapped to the yeast genome, followed by *T_m_* estimation using a previously published computational procedure [37]. Briefly, the data were normalized to the library sizes estimated by PossionSeq [80] and then fitted to an adaptive regression model to search for sharp transitions in read numbers at each probed base as a function of temperature [37]. We then averaged *T_m_* for all nucleotides with the necessary information to represent the average thermostability of a yeast mRNA, leading to estimates for 1329 and 2215 distinct yeast mRNAs in PARTE and DMS-seq data, respectively.

### The functional importance of genes

We used three different metrics as proxies for the functional importance of genes, namely, gene indispensability, mRNA expression level, and evolutionary conservation. For gene indispensability, fitness measurements of 4218 yeast strains with single-gene deletions were downloaded from a previous study by Steinmetz and colleagues [40], and the essentiality of mouse protein-coding genes was extracted from a previous study by Pal and colleagues [81]. Expression levels of mRNAs measured by RNA-seq in yeast and mouse were downloaded from GEO (GEO: GSE11209 for yeast [82] and GSE93619 [83] for mouse), to match the cell line/tissue used in RPL/PARIS. Half-lives of yeast mRNAs were extracted from a previous publication [42]. Evolutionary conservation was estimated inversely by the ratio between the number of nonsynonymous substitutions per nonsynonymous site (dN) and the number of synonymous substitutions per synonymous site (dS) detected from one-to-one orthologs between *S. cerevisiae* and *S. bayanus* following previously described pipelines [41]. Briefly, orthologous proteins were identified by reciprocal best hits of BLASTP searches between the proteomes of the two species, with the criteria of an E value <1 × 10^−20^, and alignment covering at least 80% of both orthologous sequences and a length of at least 30 amino acids. To avoid the influence of gene duplication, we used only one-to-one orthologous proteins, *i.e.*, we excluded any protein from a species that was the best hit for more than one protein in the other species. The orthologous gene pairs were realigned by ClustalW [84], filtered for gaps in alignment, and processed by PAML [85] to calculate dN/dS.

### Evolutionary conservation of each nucleotide in the yeast transcriptome

To estimate the evolutionary rates of individual sites in *S. cerevisiae* mRNA, we collected the mRNA sequences from five other post-WGD fungal species (*S. paradoxus*, *S. mikatae*, *S. bayanus*, *C. glabrata*, and *S. castellii*), along with gene orthology/paralogy information among the six species from the Fungal Orthogroups Repository [77]. Only one-to-one orthologs in all six species were used in our analysis. We aligned orthologous mRNA sequences using ClustalW [84], excluding any alignment columns with gaps in any sequence. We then used GAMMA [86] to estimate the site-specific substitution rates of each nucleotide in each mRNA. The evolutionary conservation of a nucleotide is the inverse of the substitution rate of the nucleotide.

### Odds ratios and Mantel-Haenszel test

We defined and computed an *OR*_1_ to detect within-gene correlation between evolutionary conservation and folding specificity. To estimate the *OR*_1_, a 2 × 2 contingency table was constructed for each gene by respectively categorizing each nucleotide into one of four groups on the basis of 1) whether the folding specificity of the nucleotide is higher than the mean folding specificity of all nucleotides of the gene and 2) whether the nucleotide is more conserved than the mean level of evolutionary conservation among all nucleotides of the gene. Let the numbers of sites that fall into the four groups be: *a* (yes to both questions), *b* (yes to only question 1), *c* (yes to only question 2), and *d* (no to both questions). The number of sites in each group was increased by 1 as a pseudocount to avoid division by zero. We then calculated *OR*_1_ = *ad*/*bc*. Thus, *OR*_1_ is > 1 when the conserved sites of a gene tend to have high folding specificity. The function “mantelhaen.test” in R was used to combine the *OR*_1_ values from different genes and perform the Mantel-Haenszel test (Cochran-Mantel-Haenszel chi-squared test) (Figure S4). The detection of within-gene correlation between *T_m_* and folding specificity was carried out by similarly calculating another *OR*_2_, with the exception that the 2 × 2 matrix was constructed for each gene by categorizing each nucleotide into one of four types on the basis of 1) whether the folding specificity of the nucleotide is higher than the mean folding specificity of all nucleotides of the gene and 2) whether the *T_m_* of the nucleotide is higher than the mean *T_m_* of all nucleotides of the gene.

### Ribosome profiling

The ribosome profiling data for yeast were obtained from GEO (GEO: GSE50049) [87]. The raw reads were quality-filtered and adaptor-trimmed before being mapped onto the respective genomes. We obtained the normalized ribosome density of a nucleotide from the nucleotide coverage in Ribo-Seq divided by the average coverage of the transcript to which the nucleotide belongs. Assuming negligible ribosome drop-off, this normalized ribosome density excludes the variation in mRNA abundance and translational initiation rate and is inversely correlated with the ribosome velocity. Notably, to exclude the influence of the 5′ ribosomal “ramp” [49] on peak detection for the ribosomal density, the first 200 nucleotides of each gene were removed from our analysis.

### Selective strength of point mutations affecting mRNA folding specificity

Suppose that a mutation increases the folding specificity and consequently increases the ribosome density on *p* codons by *q*-fold. The average elongation speed of the mutant is $v^{\prime }=Lv/\left(L-p+pq\right)$, where *v* is the original speed and *L* is the gene length in terms of the number of codons. When *p* ≪ *L* and *pq* ≪ *L*, we have.

$$\Delta v=v^{\prime }-v=\frac{p-pq}{L-\left(p-pq\right)}v\approx \frac{p-pq}{L}v$$

Assuming that *p* = 1 codon, *q* = 1.2 (Figure 7A), *L* = 400 codons (average length of the yeast protein), and *v* = 20 codons/s [59], [88], we obtained Δv = −0.01 codons/s. We then estimated the fitness effect of this Δv using a previously published model [11]. Briefly, the model consists of three main components. First, by assuming a fixed number of translating ribosomes, the fitness cost of slow translational elongation is estimated by the increased time requirement for synthesis of the whole proteome for the daughter cell before cell division. Second, the quantitative relationship between elongation speed and accuracy is estimated using data from an experimental study investigating the linear trade-off between the efficiency and accuracy of tRNA selection during translation [89]. Third, the benefit of reduced translational error and/or protein misfolding is modeled by assuming that a certain fraction of mistranslated proteins are misfolded and the misfolded proteins impose a dosage-dependent fitness cost, the effect size of which is experimentally determined [75]. A detailed description of the model can be found in a previous study by Yang and colleagues [11].

## Competing interests

The authors have declared no competing interests.

### CRediT authorship contribution statement

**Gongwang Yu:** Conceptualization, Methodology, Project administration, Supervision, Writing – original draft, Writing – review & editing, Data curation, Software, Formal analysis. **Hanbing Zhu:** Software, Resources, Writing – review & editing. **Xiaoshu Chen:** Conceptualization, Methodology, Project administration, Supervision, Writing – review & editing, Funding acquisition. **Jian-Rong Yang:** Conceptualization, Methodology, Project administration, Supervision, Writing – original draft, Writing – review & editing, Funding acquisition.

## Supplementary material

Supplementary Figure S1 **Demonstration of folding specificity calculation by an imaginary example A.** Four hypothetical genes containing one or two regions with potential alternative folding partners were simulated. The folding partners within each gene are represented by blue arches, with numbers above indicating the probability of observing reads supporting a particular fold. For gene ①, both regions have alternative foldings that are equally likely, i.e., the gene has no detectable signal for folding specificity (*S* = 0). For gene ②, one of the regions shows increased folding specificity because a large number of reads (50%) support one folding, while relatively few reads (10%) support the alternative folding. Genes ③ and ④ similarly have weak and strong signals of folding specificity, respectively, but have only one region with potential alternative foldings. To mimic the RPL/PARIS experiment, 10–40 chimeric reads were randomly assigned to each gene following the probabilities over the arches as supports for the foldings. The chimeric reads were then used to estimate $S_{max}$ (**B**), $S_{obs}$ (**C**), and *S* (**D**) for each hypothetical gene. The simulation of chimeric reads and the estimation of $S_{max}$, $S_{obs}$, and *S* for each hypothetical gene were repeated 1000 times, and the means and standard errors are plotted as the bars and the error bars, respectively.

Supplementary Figure S2 **Folding specificity of yeast and mouse transcripts A.** Distribution of the folding specificity of mRNAs with at least 10 chimeric reads from RPL in yeast. **B.** Same as Figure 1B, except that the folding specificities of mouse transcripts as derived from PARIS were used. **C.** The folding specificities of mRNA estimated from two PARIS replicates in mouse were compared. All genes with at least 5 chimeric PARIS reads were used to calculate Pearson’s correlation coefficient *R* = 0.48 (*P* < 10–231). Genes with folding specificities > 0 were divided into 20 equal-sized groups, and the average folding specificity for each group in either PARIS replicate was plotted, where error bars indicate the standard errors. **D.** Distribution of the folding specificity of noncoding RNAs with at least 5 chimeric reads from RPL in yeast. **E.** Same as Figure 1C, except that the folding specificities of mouse transcripts as derived from PARIS were used. The types of noncoding RNAs presented in (E) include microRNA, ribozyme, rRNA, snoRNA, and snRNA. **F.** Distribution of the folding specificities of noncoding RNAs with at least 5 chimeric reads from PARIS in mouse. ncRNAs, noncoding RNAs.

Supplementary Figure S3 **Important genes have strong folding specificities A.** The folding specificities of essential and nonessential protein-coding genes in mouse were compared by a quantile–quantile plot. Essential genes have significantly higher folding specificities than do nonessential genes (*P* < 10^−7^, Wilcoxon rank-sum test), as evidenced by the points that remained below the gray line of *x* = *y*. **B.** To exclude the possibility that the strong folding specificity of highly expressed genes is an artifact created by the abundance of chimeric reads for highly expressed mRNA, we randomly sampled five intramolecular chimeric reads from each mRNA and recalculated the folding specificity. Such randomized downsampling was repeated 1000 in yeast, and the resulting folding specificities remained positively correlated with the mRNA expression levels in yeast. **C.** Same as Figure 4B, except that the folding specificity of mouse mRNA as derived from PARIS was used. **D.** Same as (B), except that the folding specificity of mouse mRNA as derived from PARIS was used and the randomized downsampling was repeated 100 in mouse (downsampling in mouse is too slow because of the number and lengths of the genes). **E.** Same as Figure 4C, except that the folding specificity of mouse mRNA as derived from PARIS was used.

Supplementary Figure S4 **Schematic diagram of a combined odds ratio calculation based on the Mantel-Haenszel procedure A** 2 × 2 contingency table was constructed for each gene by categorizing each nucleotide into one of four types on the basis of (i) whether the folding specificity of the nucleotide is higher than the mean folding specificity of all nucleotides of the gene and (ii) whether the nucleotide is more conserved than the mean level of evolutionary conservation among all nucleotides of the gene. Let the numbers of sites that fall into the four groups be *a* (yes to both questions), *b* (yes to only question i), *c* (yes to only question ii), and *d* (no to both questions). The number of sites in each group was increased by 1 as a pseudocount to avoid division by zero. We then calculated OR1 = *ad*/*bc*. Thus, OR1 is > 1 when the conserved sites of a gene tend to have high folding specificity. Then, the Mantel-Haenszel test was used for the combined OR1 calculated for all genes using the indicated formula. OR, odds ratio; ORF, open reading frame.

Supplementary Figure S5 **mRNA folding specificity is not dominated by thermodynamics** The local influence of thermodynamic stability in vitro on folding specificity was tested by calculating an OR2 for the within-gene correspondence between the folding specificity and Tm (see Materials and methods). However, the Tm values estimated by neither DMS-seq nor PARTE yielded significant results, suggesting that the in vitro thermostability has little effect, if any, on folding specificity, a result that is consistent with the between-gene analyses (Figure 3). Combined OR2 values estimated by the Mantel-Haenszel procedure are shown. Error bars indicate the standard error, as estimated by bootstrapping the genes 1000 times. DMS-seq, dimethyl sulfate sequencing; PARTE, parallel analysis of RNA structures with temperature elevation; OR, odds ratio.

Supplementary Figure S6 **Further evidence suggesting an association of folding specificity with translation** Average normalized ribosome density downstream of the most specifically folded nucleotides. This figure is the same as Figure 7A, except that the downstream region of the most specifically folded (focal) site is shown. RPL, RNA proximity ligation.

Supplementary Figure S7 **The elevated folding specificity is consistent with the known functional secondary structure of the Zika virus RNA genome** The folding specificity profile for the first 1500 nts of the Zika virus RNA genome is shown. The folding specificity values were smoothed by a sliding window of 21 nts, with a step size of 1 nt. Red shading indicates the known long-range interaction partners (100 nts centered at both interacting regions) between the 5′ UTR and the E protein-coding region of this secondary structure in the ZIKV genome. The horizontal gray line denotes the deviation of the folding specificity from the average by 1.96 × standard deviation.

## References

[1] Hasler D., Meister G. (2016). From tRNA to miRNA: RNA-folding contributes to correct entry into noncoding RNA pathways. FEBS Lett.

[2] Dunn E.A., Rader S.D. (2010). Secondary structure of U6 small nuclear RNA: implications for spliceosome assembly. Biochem Soc Trans.

[3] Washietl S., Hofacker I.L., Lukasser M., Huttenhofer A., Stadler P.F. (2005). Mapping of conserved RNA secondary structures predicts thousands of functional noncoding RNAs in the human genome. Nat Biotechnol.

[4] Petrov A.S., Bernier C.R., Gulen B., Waterbury C.C., Hershkovits E., Hsiao C. (2014). Secondary structures of rRNAs from all three domains of life. PLoS ONE.

[5] Martin K.C., Ephrussi A. (2009). mRNA localization: gene expression in the spatial dimension. Cell.

[6] Meisner N.C., Hackermüller J., Uhl V., Aszódi A., Jaritz M., Auer M. (2004). mRNA openers and closers: modulating AU-rich element-controlled mRNA stability by a molecular switch in mRNA secondary structure. ChemBioChem.

[7] Hollams E.M., Giles K.M., Thomson A.M., Leedman P.J. (2002). mRNA stability and the control of gene expression: implications for human disease. Neurochem Res.

[8] Tian N., Yang Y., Sachsenmaier N., Muggenhumer D., Bi J., Waldsich C. (2011). A structural determinant required for RNA editing. Nucleic Acids Res.

[9] Kertesz M., Iovino N., Unnerstall U., Gaul U., Segal E. (2007). The role of site accessibility in microRNA target recognition. Nat Genet.

[10] Gu W., Zhou T., Wilke C.O. (2010). A universal trend of reduced mRNA stability near the translation-initiation site in prokaryotes and eukaryotes. PLoS Comput Biol.

[11] Yang J.R., Chen X., Zhang J. (2014). Codon-by-codon modulation of translational speed and accuracy via mRNA folding. PLoS Biol.

[12] Burkhardt D.H., Rouskin S., Zhang Y., Li G.W., Weissman J.S., Gross C.A. (2017). Operon mRNAs are organized into ORF-centric structures that predict translation efficiency. Elife.

[13] Richter J.D., Coller J. (2015). Pausing on polyribosomes: make way for elongation in translational control. Cell.

[14] Faure G., Ogurtsov A.Y., Shabalina S.A., Koonin E.V. (2016). Role of mRNA structure in the control of protein folding. Nucleic Acids Res.

[15] Park C., Chen X., Yang J.R., Zhang J. (2013). Differential requirements for mRNA folding partially explain why highly expressed proteins evolve slowly. Proc Natl Acad Sci U S A.

[16] Li C., Qian W., Maclean C.J., Zhang J. (2016). The fitness landscape of a tRNA gene. Science.

[17] Kertesz M., Wan Y., Mazor E., Rinn J.L., Nutter R.C., Chang H.Y. (2010). Genome-wide measurement of RNA secondary structure in yeast. Nature.

[18] Flynn R.A., Zhang Q.C., Spitale R.C., Lee B., Mumbach M.R., Chang H.Y. (2016). Transcriptome-wide interrogation of RNA secondary structure in living cells with icSHAPE. Nat Protoc.

[19] Underwood J.G., Uzilov A.V., Katzman S., Onodera C.S., Mainzer J.E., Mathews D.H. (2010). FragSeq: transcriptome-wide RNA structure probing using high-throughput sequencing. Nat Methods.

[20] Ding Y., Tang Y., Kwok C.K., Zhang Y., Bevilacqua P.C., Assmann S.M. (2014). *In vivo* genome-wide profiling of RNA secondary structure reveals novel regulatory features. Nature.

[21] Ramani V., Qiu R., Shendure J. (2015). High-throughput determination of RNA structure by proximity ligation. Nat Biotechnol.

[22] Lu Z., Zhang Q.C., Lee B., Flynn R.A., Smith M.A., Robinson J.T. (2016). RNA duplex map in living cells reveals higher-order transcriptome structure. Cell.

[23] Aw J.G.A., Shen Y., Wilm A., Sun M., Lim X.N., Boon K.L. (2016). *In vivo* mapping of eukaryotic RNA interactomes reveals principles of higher-order organization and regulation. Mol Cell.

[24] Rouskin S., Zubradt M., Washietl S., Kellis M., Weissman J.S. (2014). Genome-wide probing of RNA structure reveals active unfolding of mRNA structures *in vivo*. Nature.

[25] Lemay J.F., Penedo J.C., Mulhbacher J., Lafontaine D.A. (2009). Molecular basis of RNA-mediated gene regulation on the adenine riboswitch by single-molecule approaches. Methods Mol Biol.

[26] Lipfert J., Das R., Chu V.B., Kudaravalli M., Boyd N., Herschlag D. (2007). Structural transitions and thermodynamics of a glycine-dependent riboswitch from *Vibrio cholerae*. J Mol Biol.

[27] Woodson S.A. (2000). Recent insights on RNA folding mechanisms from catalytic RNA. Cell Mol Life Sci.

[28] Graveley B.R. (2016). RNA matchmaking: finding cellular pairing partners. Mol Cell.

[29] Shannon C.E. (2001). A mathematical theory of communication. Mobile Comput Commun Rev.

[30] Tuomisto H. (2010). A diversity of beta diversities: straightening up a concept gone awry. Part 1. Defining beta diversity as a function of alpha and gamma diversity. Ecography.

[31] Chouari R., Leonard M., Bouali M., Guermazi S., Rahli N., Zrafi I. (2017). Eukaryotic molecular diversity at different steps of the wastewater treatment plant process reveals more phylogenetic novel lineages. World J Microbiol Biotechnol.

[32] Mangin B., Pouilly N., Boniface M.C., Langlade N.B., Vincourt P., Vear F. (2017). Molecular diversity of sunflower populations maintained as genetic resources is affected by multiplication processes and breeding for major traits. Theor Appl Genet.

[33] Lin S.K. (1996). Molecular diversity assessment: logarithmic relations of information and species diversity and logarithmic relations of entropy and indistinguishability after rejection of Gibbs paradox of entropy of mixing. Molecules Online.

[34] Medinger R., Nolte V., Pandey R.V., Jost S., Ottenwälder B., Schlötterer C. (2010). Diversity in a hidden world: potential and limitation of next-generation sequencing for surveys of molecular diversity of eukaryotic microorganisms. Mol Ecol.

[35] Huynen M., Gutell R., Konings D. (1997). Assessing the reliability of RNA folding using statistical mechanics. J Mol Biol.

[36] Yoshihama M., Nakao A., Kenmochi N. (2013). snOPY: a small nucleolar RNA orthological gene database. BMC Res Notes.

[37] Wan Y., Qu K., Ouyang Z., Kertesz M., Li J., Tibshirani R. (2012). Genome-wide measurement of RNA folding energies. Mol Cell.

[38] Zadeh J.N., Wolfe B.R., Pierce N.A. (2011). Nucleic acid sequence design via efficient ensemble defect optimization. J Comput Chem.

[39] Pan T., Sosnick T. (2006). RNA folding during transcription. Annu Rev Biophys Biomol Struct.

[40] Steinmetz L.M., Scharfe C., Deutschbauer A.M., Mokranjac D., Herman Z.S., Jones T. (2002). Systematic screen for human disease genes in yeast. Nat Genet.

[41] Zhang J., Yang J.R. (2015). Determinants of the rate of protein sequence evolution. Nat Rev Genet.

[42] Shalem O., Dahan O., Levo M., Martinez M.R., Furman I., Segal E. (2008). Transient transcriptional responses to stress are generated by opposing effects of mRNA production and degradation. Mol Syst Biol.

[43] Wells S.E., Hillner P.E., Vale R.D., Sachs A.B. (1998). Circularization of mRNA by eukaryotic translation initiation factors. Mol Cell.

[44] Muzzey D., Sherlock G., Weissman J.S. (2014). Extensive and coordinated control of allele-specific expression by both transcription and translation in *Candida albicans*. Genome Res.

[45] Yang J.R., Zhang J. (2015). Human long noncoding RNAs are substantially less folded than messenger RNAs. Mol Biol Evol.

[46] Yang J.R. (2017). Does mRNA structure contain genetic information for regulating co-translational protein folding?. Zool Res.

[47] Charneski C.A., Hurst L.D. (2013). Positively charged residues are the major determinants of ribosomal velocity. PLoS Biol.

[48] Diament A., Tuller T. (2016). Estimation of ribosome profiling performance and reproducibility at various levels of resolution. Biol Direct.

[49] Tuller T., Carmi A., Vestsigian K., Navon S., Dorfan Y., Zaborske J. (2010). An evolutionarily conserved mechanism for controlling the efficiency of protein translation. Cell.

[50] Yang J.R., Zhuang S.M., Zhang J. (2010). Impact of translational error-induced and error-free misfolding on the rate of protein evolution. Mol Syst Biol.

[51] O'Brien E.P., Vendruscolo M., Dobson C.M. (2012). Prediction of variable translation rate effects on cotranslational protein folding. Nat Commun.

[52] Wagner A. (2005). Energy constraints on the evolution of gene expression. Mol Biol Evol.

[53] Park L. (2011). Effective population size of current human population. Genet Res (Camb).

[54] Jungfleisch J., Nedialkova D.D., Dotu I., Sloan K.E., Martinez Bosch N., Bruning L. (2017). A novel translational control mechanism involving RNA structures within coding sequences. Genome Res.

[55] Gruber A.R., Lorenz R., Bernhart S.H., Neuböck R., Hofacker I.L. (2008). The Vienna RNA website. Nucleic Acids Res.

[56] Li P., Wei Y., Mei M., Tang L., Sun L., Huang W. (2018). Integrative analysis of zika virus genome RNA structure reveals critical determinants of viral infectivity. Cell Host Microbe.

[57] Pörschke D. (1974). Thermodynamic and kinetic parameters of an oligonucleotide hairpin helix. Biophys Chem.

[58] Ingolia N.T., Lareau L.F., Weissman J.S. (2011). Ribosome profiling of mouse embryonic stem cells reveals the complexity and dynamics of mammalian proteomes. Cell.

[59] Gilchrist M.A., Wagner A. (2006). A model of protein translation including codon bias, nonsense errors, and ribosome recycling. J Theor Biol.

[60] Metzger B.P.H., Yuan D.C., Gruber J.D., Duveau F., Wittkopp P.J. (2015). Selection on noise constrains variation in a eukaryotic promoter. Nature.

[61] Yang J.R., Liao B.Y., Zhuang S.M., Zhang J. (2012). Protein misinteraction avoidance causes highly expressed proteins to evolve slowly. Proc Natl Acad Sci U S A.

[62] Liu Z., Zhang J. (2017). Human C-to-U coding RNA editing is largely nonadaptive. Mol Biol Evol.

[63] Liu Z., Zhang J. (2017). Most m^6^A RNA modifications in protein-coding regions are evolutionarily unconserved and likely nonfunctional. Mol Biol Evol.

[64] Xu G., Zhang J. (2014). Human coding RNA editing is generally nonadaptive. Proc Natl Acad Sci U S A.

[65] Park C., Zhang J. (2011). Genome-wide evolutionary conservation of *N*-glycosylation sites. Mol Biol Evol.

[66] Mao Y., Liu H.L., Liu Y., Tao S. (2014). Deciphering the rules by which dynamics of mRNA secondary structure affect translation efficiency in *Saccharomyces cerevisiae*. Nucleic Acids Res.

[67] Arava Y., Wang Y., Storey J.D., Liu C.L., Brown P.O., Herschlag D. (2003). Genome-wide analysis of mRNA translation profiles in *Saccharomyces cerevisiae*. Proc Natl Acad Sci U S A.

[68] Gralla J., Crothers D.M. (1973). Free energy of imperfect nucleic acid helices. 3. Small internal loops resulting from mismatches. J Mol Biol.

[69] Qi F., Frishman D. (2017). Melting temperature highlights functionally important RNA structure and sequence elements in yeast mRNA coding regions. Nucleic Acids Res.

[70] O'Brien E.P., Vendruscolo M., Dobson C.M. (2014). Kinetic modelling indicates that fast-translating codons can coordinate cotranslational protein folding by avoiding misfolded intermediates. Nat Commun.

[71] Shalgi R., Hurt J.A., Krykbaeva I., Taipale M., Lindquist S., Burge C.B. (2013). Widespread regulation of translation by elongation pausing in heat shock. Mol Cell.

[72] Shabalina S.A., Spiridonov N.A., Kashina A. (2013). Sounds of silence: synonymous nucleotides as a key to biological regulation and complexity. Nucleic Acids Res.

[73] Beaudoin J.D., Novoa E.M., Vejnar C.E., Yartseva V., Takacs C.M., Kellis M. (2018). Analyses of mRNA structure dynamics identify embryonic gene regulatory programs. Nat Struct Mol Biol.

[74] Takyar S., Hickerson R.P., Noller H.F. (2005). mRNA helicase activity of the ribosome. Cell.

[75] Drummond D.A., Wilke C.O. (2008). Mistranslation-induced protein misfolding as a dominant constraint on coding-sequence evolution. Cell.

[76] Aken B.L., Achuthan P., Akanni W., Amode M.R., Bernsdorff F., Bhai J. (2017). Ensembl 2017. Nucleic Acids Res.

[77] Wapinski I., Pfeffer A., Friedman N., Regev A. (2007). Automatic genome-wide reconstruction of phylogenetic gene trees. Bioinformatics.

[78] Barrett T., Wilhite S.E., Ledoux P., Evangelista C., Kim I.F., Tomashevsky M. (2013). NCBI GEO: archive for functional genomics data sets—update. Nucleic Acids Res.

[79] Dobin A., Davis C.A., Schlesinger F., Drenkow J., Zaleski C., Jha S. (2013). STAR: ultrafast universal RNA-seq aligner. Bioinformatics.

[80] Li J., Witten D.M., Johnstone I.M., Tibshirani R. (2012). Normalization, testing, and false discovery rate estimation for RNA-sequencing data. Biostatistics.

[81] Pál C., Papp B., Hurst L.D. (2003). Genomic function: rate of evolution and gene dispensability. Nature.

[82] Nagalakshmi U., Wang Z., Waern K., Shou C., Raha D., Gerstein M. (2008). The transcriptional landscape of the yeast genome defined by RNA sequencing. Science.

[83] Good C.R., Madzo J., Patel B., Maegawa S., Engel N., Jelinek J. (2017). A novel isoform of TET1 that lacks a CXXC domain is overexpressed in cancer. Nucleic Acids Res.

[84] Thompson J.D., Gibson T.J., Higgins D.G. (2002). Multiple sequence alignment using ClustalW and ClustalX. Curr Protoc Bioinformatics.

[85] Yang Z. (2007). PAML 4: phylogenetic analysis by maximum likelihood. Mol Biol Evol.

[86] Gu X., Zhang J. (1997). A simple method for estimating the parameter of substitution rate variation among sites. Mol Biol Evol.

[87] Artieri C.G., Fraser H.B. (2014). Evolution at two levels of gene expression in yeast. Genome Res.

[88] von der Haar T. (2008). A quantitative estimation of the global translational activity in logarithmically growing yeast cells. BMC Syst Biol.

[89] Johansson M., Zhang J., Ehrenberg M. (2012). Genetic code translation displays a linear trade-off between efficiency and accuracy of tRNA selection. Proc Natl Acad Sci U S A.

