# Supplementary figures and images for: Specificity of mRNA Folding and Its Association with Evolutionarily Adaptive mRNA Secondary Structures
Source: Genomics Proteomics Bioinformatics. 2021 Feb 17;19(6):882–900. doi: 10.1016/j.gpb.2019.11.013 (PMC9403030; doi:10.1016/j.gpb.2019.11.013)

**A**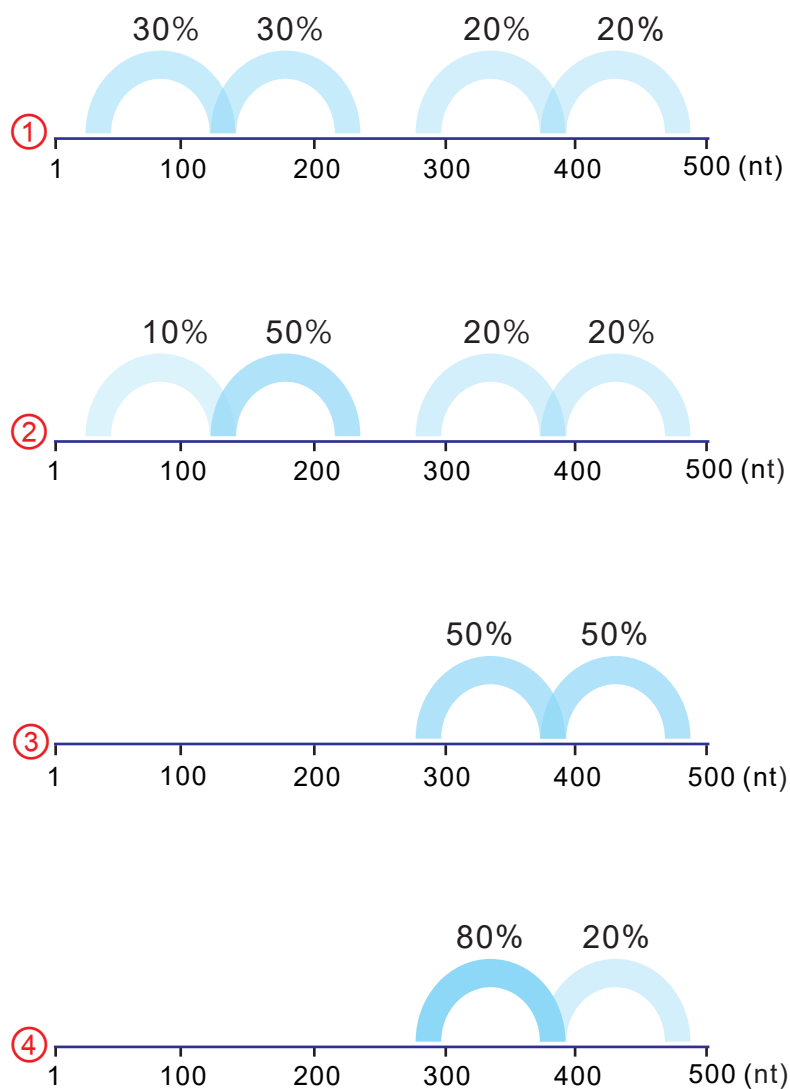**B**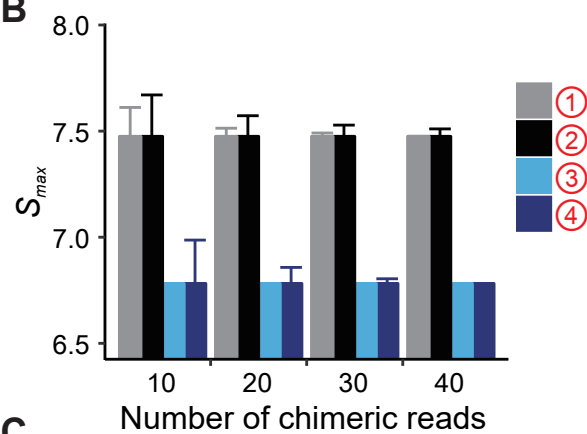**C**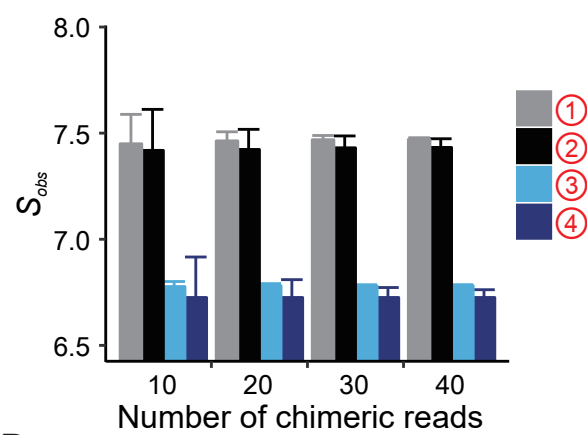**D**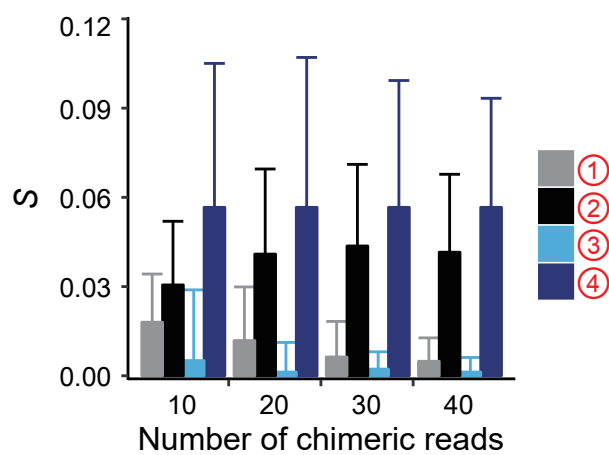

Supplement: Supplementary Figure S1 — Demonstration of folding specificity calculation by an imaginary example A. Four hypothetical genes containing one or two regions with potential alternative folding partners were simulated. The folding partners within each gene are represented by blue arches, with numbers above indicating the probability of observing reads supporting a particular fold. For gene ①, both regions have alternative foldings that are equally likely, i.e., the gene has no detectable signal for folding specificity (S = 0). For gene ②, one of the regions shows increased folding specificity because a large number of reads (50%) support one folding, while relatively few reads (10%) support the alternative folding. Genes ③ and ④ similarly have weak and strong signals of folding specificity, respectively, but have only one region with potential alternative foldings. To mimic the RPL/PARIS experiment, 10–40 chimeric reads were randomly assigned to each gene following the probabilities over the arches as supports for the foldings. The chimeric reads were then used to estimate Smax (B), Sobs (C), and S (D) for each hypothetical gene. The simulation of chimeric reads and the estimation of Smax, Sobs, and S for each hypothetical gene were repeated 1000 times, and the means and standard errors are plotted as the bars and the error bars, respectively. [file mmc1.pdf]

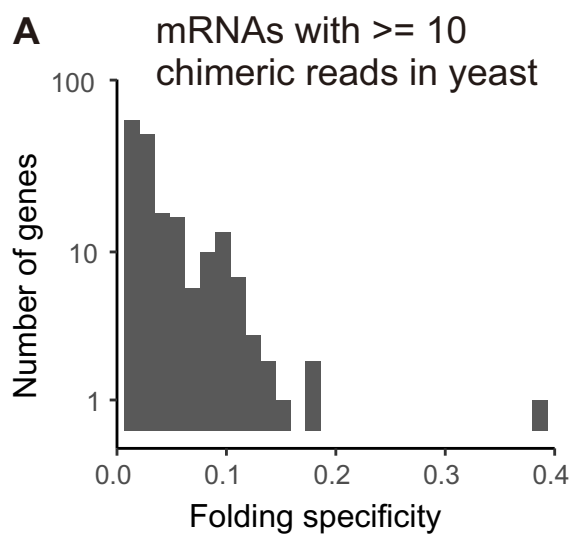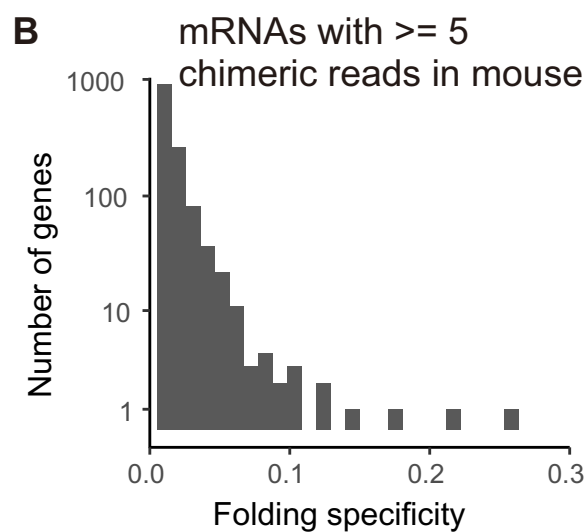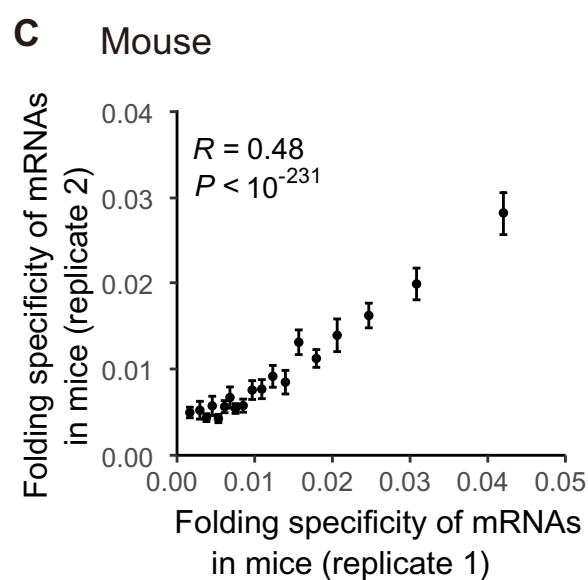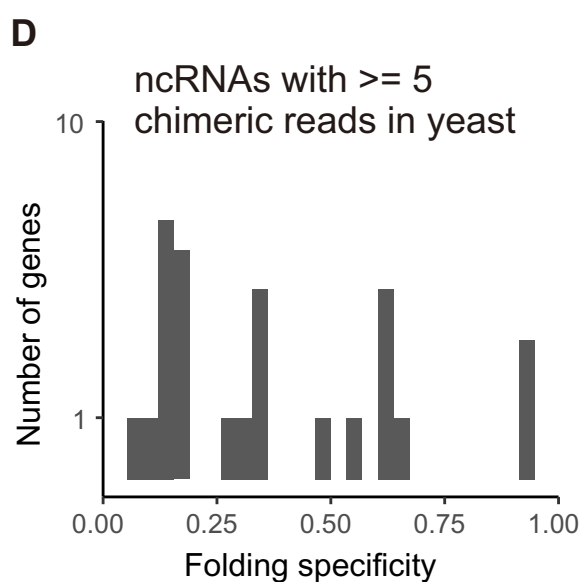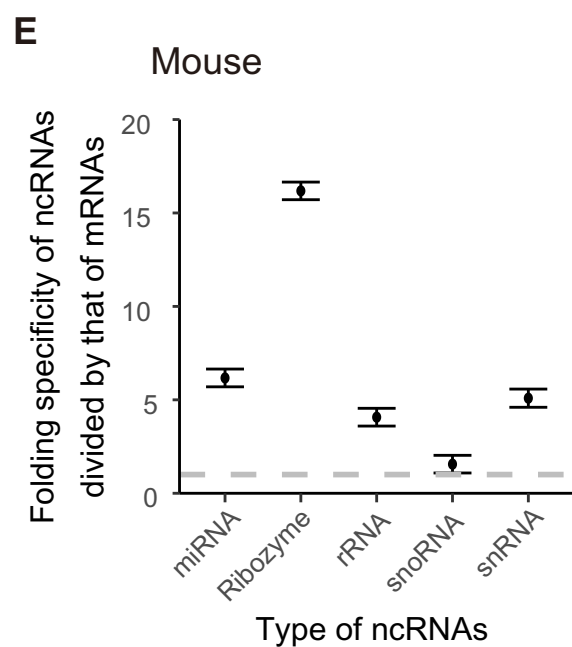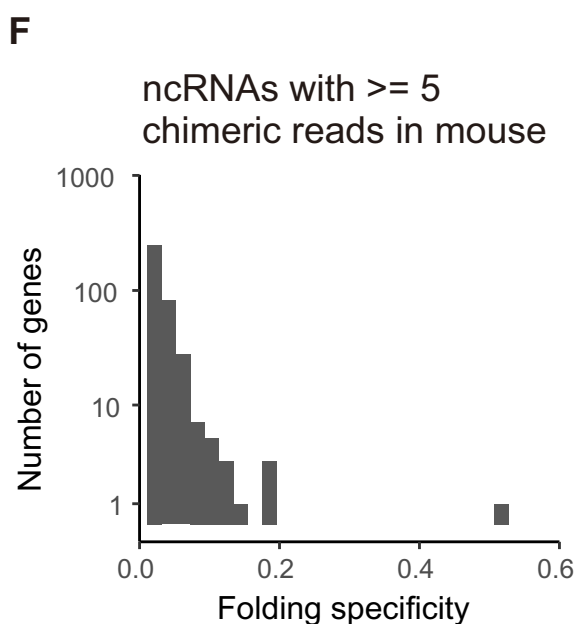

Supplement: Supplementary Figure S2 — Folding specificity of yeast and mouse transcripts A. Distribution of the folding specificity of mRNAs with at least 10 chimeric reads from RPL in yeast. B. Same as Figure 1B, except that the folding specificities of mouse transcripts as derived from PARIS were used. C. The folding specificities of mRNA estimated from two PARIS replicates in mouse were compared. All genes with at least 5 chimeric PARIS reads were used to calculate Pearson’s correlation coefficient R = 0.48 (P < 10–231). Genes with folding specificities > 0 were divided into 20 equal-sized groups, and the average folding specificity for each group in either PARIS replicate was plotted, where error bars indicate the standard errors. D. Distribution of the folding specificity of noncoding RNAs with at least 5 chimeric reads from RPL in yeast. E. Same as Figure 1C, except that the folding specificities of mouse transcripts as derived from PARIS were used. The types of noncoding RNAs presented in (E) include microRNA, ribozyme, rRNA, snoRNA, and snRNA. F. Distribution of the folding specificities of noncoding RNAs with at least 5 chimeric reads from PARIS in mouse. ncRNAs, noncoding RNAs. [file mmc2.pdf]

**A Mouse**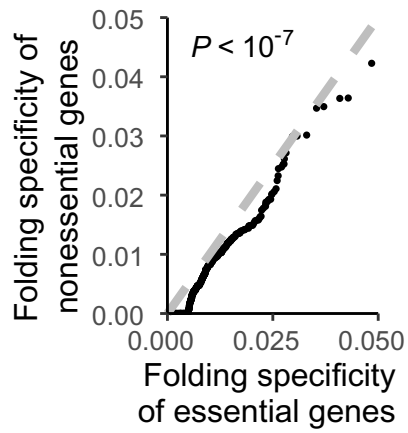**B Yeast**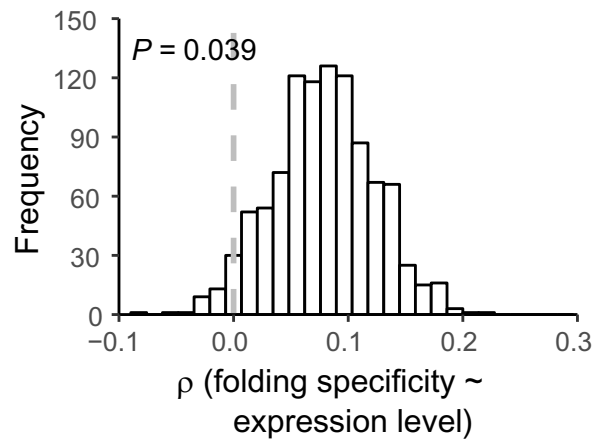**C Mouse**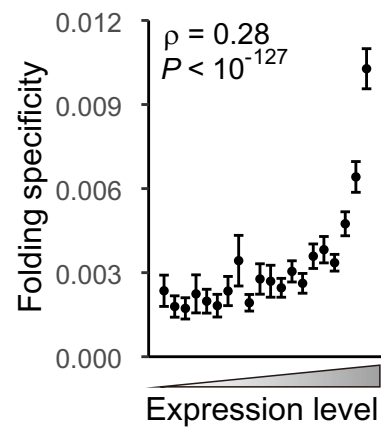**D Mouse**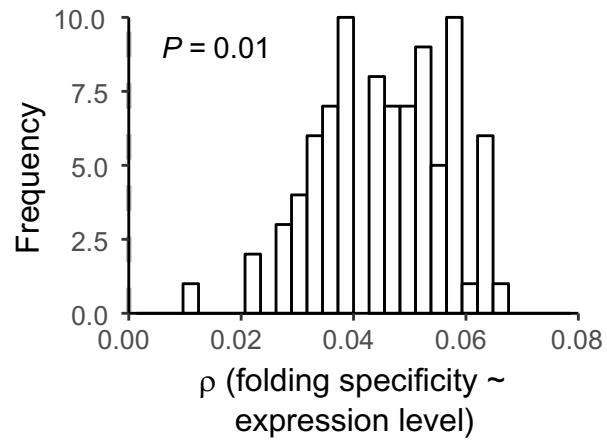**E Mouse**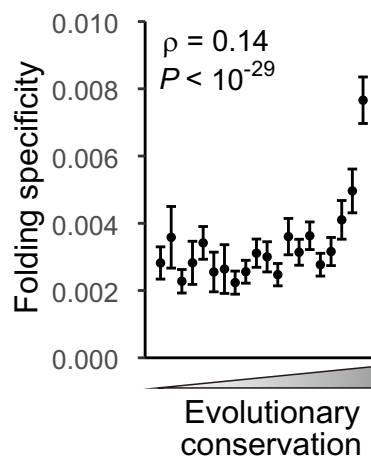

Supplement: Supplementary Figure S3 — Important genes have strong folding specificities A. The folding specificities of essential and nonessential protein-coding genes in mouse were compared by a quantile–quantile plot. Essential genes have significantly higher folding specificities than do nonessential genes (P < 10−7, Wilcoxon rank-sum test), as evidenced by the points that remained below the gray line of x = y. B. To exclude the possibility that the strong folding specificity of highly expressed genes is an artifact created by the abundance of chimeric reads for highly expressed mRNA, we randomly sampled five intramolecular chimeric reads from each mRNA and recalculated the folding specificity. Such randomized downsampling was repeated 1000 in yeast, and the resulting folding specificities remained positively correlated with the mRNA expression levels in yeast. C. Same as Figure 4B, except that the folding specificity of mouse mRNA as derived from PARIS was used. D. Same as (B), except that the folding specificity of mouse mRNA as derived from PARIS was used and the randomized downsampling was repeated 100 in mouse (downsampling in mouse is too slow because of the number and lengths of the genes). E. Same as Figure 4C, except that the folding specificity of mouse mRNA as derived from PARIS was used. [file mmc3.pdf]

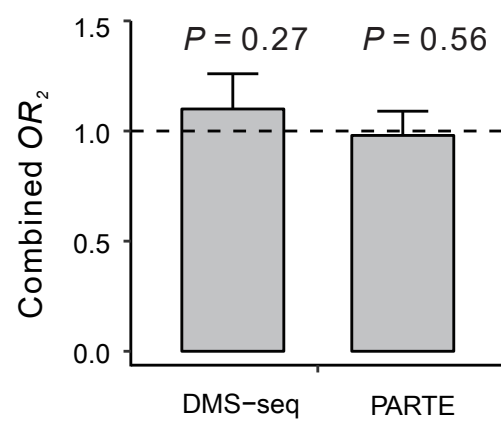

Supplement: Supplementary Figure S5 — mRNA folding specificity is not dominated by thermodynamics The local influence of thermodynamic stability in vitro on folding specificity was tested by calculating an OR2 for the within-gene correspondence between the folding specificity and Tm (see Materials and methods). However, the Tm values estimated by neither DMS-seq nor PARTE yielded significant results, suggesting that the in vitro thermostability has little effect, if any, on folding specificity, a result that is consistent with the between-gene analyses (Figure 3). Combined OR2 values estimated by the Mantel-Haenszel procedure are shown. Error bars indicate the standard error, as estimated by bootstrapping the genes 1000 times. DMS-seq, dimethyl sulfate sequencing; PARTE, parallel analysis of RNA structures with temperature elevation; OR, odds ratio. [file mmc5.pdf]

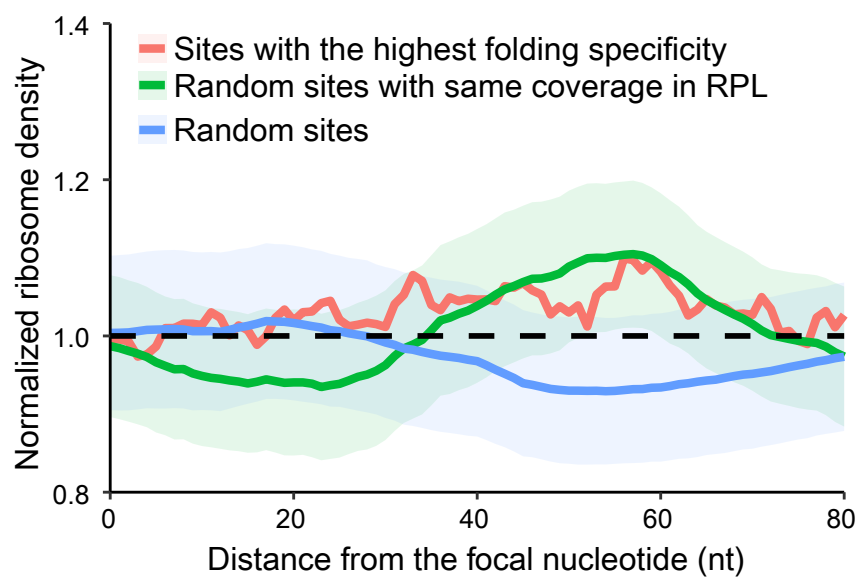

Supplement: Supplementary Figure S6 — Further evidence suggesting an association of folding specificity with translation Average normalized ribosome density downstream of the most specifically folded nucleotides. This figure is the same as Figure 7A, except that the downstream region of the most specifically folded (focal) site is shown. RPL, RNA proximity ligation. [file mmc6.pdf]

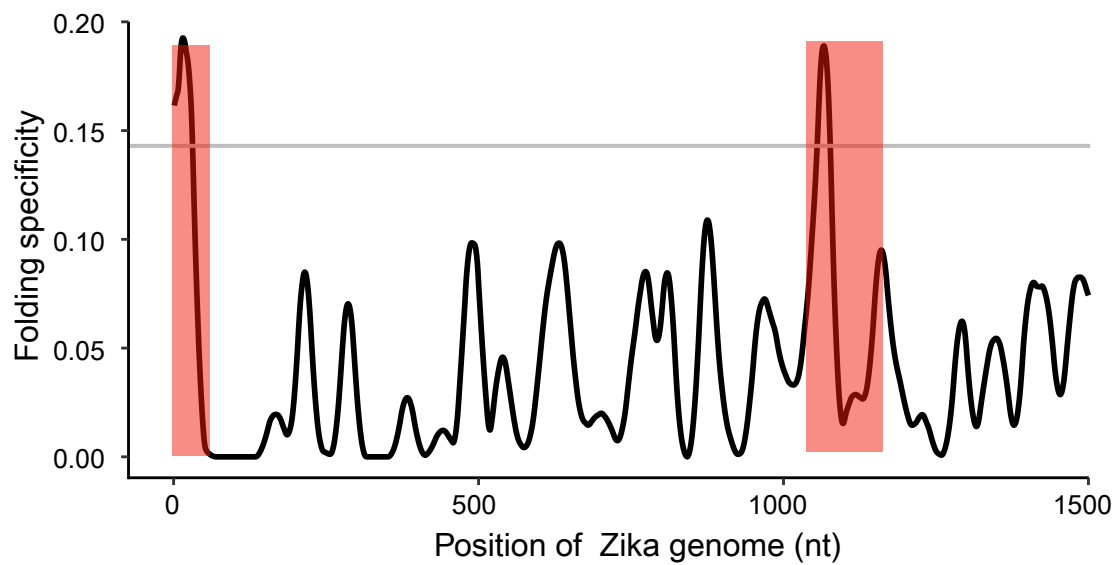

Supplement: Supplementary Figure S7 — The elevated folding specificity is consistent with the known functional secondary structure of the Zika virus RNA genome The folding specificity profile for the first 1500 nts of the Zika virus RNA genome is shown. The folding specificity values were smoothed by a sliding window of 21 nts, with a step size of 1 nt. Red shading indicates the known long-range interaction partners (100 nts centered at both interacting regions) between the 5′ UTR and the E protein-coding region of this secondary structure in the ZIKV genome. The horizontal gray line denotes the deviation of the folding specificity from the average by 1.96 × standard deviation. [file mmc7.pdf]
