# Supplementary material for: Specificity of mRNA Folding and Its Association with Evolutionarily Adaptive mRNA Secondary Structures
Source: Genomics Proteomics Bioinformatics. 2021 Feb 17;19(6):882–900. doi: 10.1016/j.gpb.2019.11.013 (PMC9403030; doi:10.1016/j.gpb.2019.11.013)
Supplement: Supplementary Figure S4 — Schematic diagram of a combined odds ratio calculation based on the Mantel-Haenszel procedure A 2 × 2 contingency table was constructed for each gene by categorizing each nucleotide into one of four types on the basis of (i) whether the folding specificity of the nucleotide is higher than the mean folding specificity of all nucleotides of the gene and (ii) whether the nucleotide is more conserved than the mean level of evolutionary conservation among all nucleotides of the gene. Let the numbers of sites that fall into the four groups be a (yes to both questions), b (yes to only question i), c (yes to only question ii), and d (no to both questions). The number of sites in each group was increased by 1 as a pseudocount to avoid division by zero. We then calculated OR1 = ad/bc. Thus, OR1 is > 1 when the conserved sites of a gene tend to have high folding specificity. Then, the Mantel-Haenszel test was used for the combined OR1 calculated for all genes using the indicated formula. OR, odds ratio; ORF, open reading frame. [file mmc4.pdf]

ORF: *YGR180C*		Folding specificity	
$n_{YGR180C}$ = 547 , $OR_{1\ YGR180C}$ = 1.52		> Mean (within gene)	<= Mean (within gene)
Conservation across 6 species	Conserved	$a_{YGR180C}$ = 109	$c_{YGR180C}$ = 82
	Not conserved	$b_{YGR180C}$ = 166	$d_{YGR180C}$ = 190

ORF: *YNL160W*		Folding specificity	
$n_{YNL160W}$ = 362 , $OR_{1\ YNL160W}$ = 1.49		> Mean (within gene)	<= Mean (within gene)
Conservation across 6 species	Conserved	$a_{YNL160W}$ = 29	$c_{YNL160W}$ = 19
	Not conserved	$b_{YNL160W}$ = 159	$d_{YNL160W}$ = 155

$$OR_{1\,g} = \frac{a_g\, d_g}{b_g\, c_g}$$

$$\text{Mantel-Haenszel test : combined } OR_1 = \frac{\sum_{g \in G} a_g d_g / n_g}{\sum_{g \in G} b_g c_g / n_g}$$
